# Between land and sea: A multidisciplinary approach to understand the Early Occupation of Sicily (EOS)

**DOI:** 10.1371/journal.pone.0299118

**Published:** 2024-10-09

**Authors:** Ilaria Patania, Isaac Ogloblin Ramirez, Peyton Carroll, Kristen Wroth, Sara Zaia, Sebastiano Di Mauro, Danielle Falci, Iris Querenet Onfroy de Breville, Ignacio Aguilar Lazagabaster, Gianmarco Minniti, Agatino Reitano, Gianni Insacco, Tristram Kidder, Christian Tryon

**Affiliations:** 1 Department of Anthropology, Washington University in St. Louis, MO, United States of America; 2 Center for Human Evolutionary Studies, Rutgers University, NJ, United States of America; 3 Department of Maritime Civilizations, University of Haifa, Haifa, Israel; 4 The Leon Recanati Institute for Maritime Studies, Haifa, Israel; 5 The Sonia and Marco Nadler Institute of Archeology, Tel Aviv University, Tel Aviv, Israel; 6 Department of Anthropology, University of Connecticut, Storrs, CT, United States of America; 7 Department of Chemistry, Earlham College, Richmond, IN, United States of America; 8 Department of Anthropology, Harvard University, Cambridge, MA, United States of America; 9 Independent Scholar, Augusta, Italy; 10 National Research Center on Human Evolution, Burgos, Spain; 11 Department of Evolution, Ecology & Behaviour, University of Liverpool, Liverpool, United Kingdom; 12 Dipatimento di Geologia, Università di Catania, Catania, Italy; 13 Museo Civico di Storia Naturale di Comiso, Comiso, Italy; 14 Human Origins Program, National Museum of Natural History, Smithsonian Institution, Washington DC, United States of America; Sapienza University of Rome: Universita degli Studi di Roma La Sapienza, ITALY

## Abstract

The island of Sicily is considered to be among the first occupied by humans in the European Upper Paleolithic. Studies to understand early occupation of the island are mostly concentrated on the northern shores. Our project, Early Occupation of Sicily (EOS), focuses on southeastern Sicily so to address questions regarding dispersal to Mediterranean islands and Late Pleistocene landscapes and environments. Here, we present the initial results of our terrestrial and underwater surveys in combination with archival work and analyses of museum collections. In SE Sicily very few Upper Paleolithic sites have been excavated and analyzed using scientific methods. We have relocated and assessed ~20 caves and rock shelters identified between the 1870s and 1990s, studied museum collections, and collected raw material to reconstruct procurement patterns. To identify new sites, we conducted land and underwater surveys to reconstruct paleo-shorelines and past environments. We have identified three sites, two on land and one partially submerged, that still contain unexplored archaeological sediments, demonstrated in one instance through seismic tomography. This work shows the potential of re-examining minimally studied sites and materials to reconstruct mobility patterns and environmental impact of the first inhabitants of the island.

## Introduction

Despite the long history of research in the Mediterranean our understanding of human dispersal and early occupation of its islands is still sparse, and highly debated, especially for sites that predate the Holocene [1]. The absence of conclusive evidence for the pre-LGM human occupation of the Mediterranean islands has previously been attributed to the low trophic level on some of the smaller islands, which may not have been able to support a population. To fully establish habitation on the Mediterranean islands, the role of domesticated animals may have been crucial [1]. Recent hypotheses by Slimak [2] have highlighted the potential for crossing the Mediterranean as an important mechanism for the expansion of *Homo sapiens* into southwestern Asia, causing us to reconsider the role of islands in this process. On biogeographical grounds, because of its proximity to the mainland and relatively large size (25,711 km^2^), Sicily is a likely candidate for hosting the earliest human occupation among the Mediterranean islands [1]. However, here and elsewhere in the Mediterranean, uncontroversial evidence for human arrival, whether by land bridge or seafaring, is placed at ~16 kya shortly after the Last Glacial Maximum (LGM ~26.5–19 ka) [1, 3, 4] (Table 1), which is quite late when considering the much earlier keystone dates for the dispersal of *H*. *sapiens*, such as dispersal by land into Siberia by 45 ka [5] and by open-ocean crossings eastward to Australia between 65 and 46 ka [6].

**Table 1:** Complete list of published Paleolithic radiometric dates for Sicilian sites with references.

Site	Strata	Lab No.	14C Date	cal BP 2*σ*	Source
Acqua Fitusa^¶^	B	F-26	13760 ± 330	17,740–15,550	[8]
Acqua Fitusa	B, tgl. 3	Beta-194335	11440 ± 50	17,676–17,659*	[9] originally in [10]
Addaura Caprara	Individual 1	KIA-36055	12890 ± 60	15,950–15,000	[3]
Castello	3	OxA-9975	11070 ± 130	13,174–12,751*	[11]
Castello	3	OxA-9976	10030 ± 130	11,990–11,985*	[11]
Castello	3	OxA-10105	10520 ± 65	12,708–12,432*	[11]
Castello	2C2	OxA-9977	9580 ± 160	11,270–10,485*	[11]
Castello	2C1	OxA-9978	11350 ± 100	13,136–13,124*	[11]
Castello	2C1	OxA-9979	11350 ± 180	13,586–13,546*	[11]
Castello	2B	OxA-10002	12670 ± 65	15,307–14,897*	[11]
Castello	2B	OxA-10003	12800 ± 60	15,524–15,096*	[11]
Castello	1C	OxA-10037	12855 ± 70	15,594–15,149*	[11]
Castello	1C	OxA-10038	12975 ± 70	15,738–15,289*	[11]
Castello	1A2	OxA-10039	13265 ± 70	16,169–15,717*	[11]
Castello	1A1	OxA-10040	13485 ± 80	16,920–16,230	[3]
d’Oriente	7D	LTL-14260A	12149 ± 65	14,136–13,932	[12]
d’Oriente	7E	LTL-873A	12132 ± 80	14,210–13,770	[3]
Giovanna^¶^	II	R-484	12840 ± 100	16,150–14,890	[3]
Incisioni^†^ (Addaura)	Trench III, spit 13	OxA-13818	12165 ± 50	13,628–13,203*	[9]
Incisioni^†^ (Addaura)	Trench III, spit 1	OxA-13819	12310 ± 50	13,791–13,363*	[9]
Incisioni^†^ (Addaura)	Trench III, spit 2	OxA-13820	12480 ± 45	14,040–12,650	[3]
Incisioni^†^ (Addaura)	Trench III, spit 2	OxA-13821	12455 ± 50	14001–13526*	[9]
Incisioni^†^ (Addaura)	ADD-8	AAR-26308	11023 ± 46	12,648–12,229	[13]
Incisioni^†^ (Addaura)	ADD-11	AAR-26309	12266 ± 53	13,747–13,542	[13]
Incisioni^†^ (Addaura)	ADD-15	AAR-26310	12763 ± 48	14,384–14,071	[13]
Incisioni^†^ (Addaura)	ADD-16	AAR-26311	10724 ± 43	12,136–11,849	[13]
Incisioni^†^ (Addaura)	ADD-18	AAR-30174	12066 ± 47	13,510–13,335	[13]
Levanzo^¶^^†^ (Genovesi)	Spit 6A, lower	F-19	11710 ± 295	13,740–12,590	[9]
Levanzo^¶^^†^ (Genovesi)	Spit 6, lower	F-20	10110 ± 300	11,827–10,109*	[9]
Levanzo^¶^^†^ (Genovesi)	Spit 5A, lower	F-18	10175 ± 300	11,908–10,183*	[9]
Levanzo^¶^^†^ (Genovesi)	3	R-566	11180 ± 120	12,729–12,048*	[9]
Levanzo^†^ (Genovesi)	Spit 10	OxA-14258	10750 ± 45	12,166–11,555*	[9]
Levanzo^†^ (Genovesi)	Spit 12	OxA-14259	11200 ± 45	12,689–12,261*	[9]
Niscemi^†^	Spit 6	OxA-13823	12090 ± 50	13,526–13,141*	[9]
Niscemi^†^	Spit 8	OxA-13824	12440 ± 50	14,010–13,630	[9]
Niscemi^†^	Spit 10	OxA-14255	11170 ± 45	12,666–12,205*	[9]
Perciata^¶^^†^		F-27	11960 ± 330	14,090–12,650	[3]
San Teodoro^¶^	B, facies B1	DSH-2749	18330 ± 400	23,000–20,910	[3]
San Teodoro	PAL	DSH9270-GE	12624 ± 59	15,224–14,708	[14]
San Teodoro	Individual 1	ETH-34451	12580 ± 130	15,240–14,120	[3]
Schiacciata^†^	XII	OxA-15561	12355 ± 50	13,900–13,470	[3]
Uccerie	4D	LTL-1517A	13191 ± 120	16,660–15,250	[3]
Uccerie	4E	LTL-1518A	12933 ± 75	15,696–15,237*	[15]
Uccerie	4C	LTL-1516A	12958 ± 90	15,764–15,236*	[15]

Alternative site names are given in parentheses. ¶ Non-AMS Date.

† Date from marine shell.

* Dates calibrated by authors using the original uncalibrated data reported by excavators. Calibrations were done using the rcarbon R package with the IntCal20 [7] calibration curve (terrestrial) and the Marine20 calibration curve for marine shells corrected using the local reservoir age value for Sicily [3].

Although hypothesized, an African route into Sicily for Upper Palaeolithic (UP) humans seems unlikely [reviewed in 4, 15, 16]. Instead, based on broad similarities in lithic technology (i.e., Epigravettian), cranial morphology [17] and ancient DNA [2, 18], most scholars agree that mainland Italy is the most likely source for the UP dispersion into the island [1, 15]. Human, and certainly animal migration, was most likely facilitated by a land bridge that connected Sicily to Italy during the last glaciation [3]. In fact, early human occupation in Sicily appears broadly coincident with a period of major faunal change and in the introduction of several large and small mammals. Palaeontological analyses [19, 20] arranged the Pleistocene vertebrates into five Faunal Complexes (FC). The fifth, Castello FC, dating from the Late Pleistocene, contains extant continental species together with paleolithic artifacts, is made up of continental European taxa including European wild ass (*Equus hydruntinus*), red deer (*Cervus elaphus*), auroch (*Bos primigenius*), wild boar (*Sus scrofa*), and carnivores such the weasel (*Mustela sp*.) and fox (*Vulpes vulpes*). The Castello FC replaces mammals of the previous FC of the late Middle Pleistocene/Late Pleistocene, such the ~2 m-tall elephant *Palaeoloxodon mnaidriensis*, the cave hyena (*Crocuta crocuta spelaea*) and a number of endemic ungulates. However, the timing and dynamics of this replacement is not yet clear.

The relationships, if any, between human occupation, and environmental change/faunal extinction are unclear largely because of the rarity of archaeological sites with carefully excavated and finely resolved records. This paucity of research affects also our understanding of Late Glacial and post-Glacial environments, and in general, paleoenvironmental records during and immediately following the LGM are rare, with a marked unconformity in cores from Lake Pergusa [21]; pollen or other evidence for interpreting terrestrial environments also are absent from the Lago Preola cores [22]. Faunal data are generally sparse, and primarily focused on taxonomic identification (Table 2) rather than zooarchaeological investigations of human hunting or butchery practices for Epigravettian sites, although the situation improves substantially for the Holocene Mesolithic record [e.g., 23, 24].

**Table 2:** Summary faunal data for Epigravettian sites from Sicily. Data from [1,5, 23, 25–30].

Species	Common name	Levanzo/Genovesi Strato 3, tagli 5 & 6	Uccerie Strato 4D	Oriente Strato 7	Uzzo Strato Basale 33–48	Castello	San Teodoro Layer D	Pedagaggi	Corruggi	Giovanna	San Corrado
**Ungulates**											
*Sus scrofa*	wild boar	4	41	2	6	5	26	12	6	Present	Present
*Cervus elaphus*	red deer	129	6	104	0	52	127	11	87	Present	
*Cervus* sp.	deer				357			1			Present
*Bos primigenius†*	auroch	32	28	9	10	9	49	7	2	Present	
*Bos* sp.	bovid								5		
*Equus hydruntinus†*	wild ass	17	57	14	0	52		15	8	Present	
*Equus* cf. *hydruntinus*	probable wild ass										Present
**Carnivores**											
*Vulpes vulpes*	red fox	6	3	11	6	5	1		43	Present	
cf. *Lynx lynx*	lynx							1			
*Felis sylvestris*	European wildcat								7		
*Canis* cf. *lupus*	wolf				3				2	Present	
*Ursus* sp.	bear				1						
*Martes* sp.	martin				1						
*Mustela* cf. *nivalis*	least weasel				2						
**Small mammals**											
*Arvicola terrestris*	water vole	2	0	0							
*Lepus* sp.	hare	0	1	0		1					
*Erinaceus europaeus*	European hedgehog	0	0	11	6	1			1		
*Microtus* sp.	vole				7				2		
*Apodemus* sp.	mouse				1						
*Oryctolagus cuniculus*	European rabbit								13		
NISP		190	136	151	400	125	203	47	176	0	0
Ntaxa		6	6	6	10	6	4	5	9		
Equid: Deer ratio		0.13	9.50	0.13	0.00	1.00	0.00	1.36	0.09	N/A	N/A

†Extinct.

**Fig 1:**
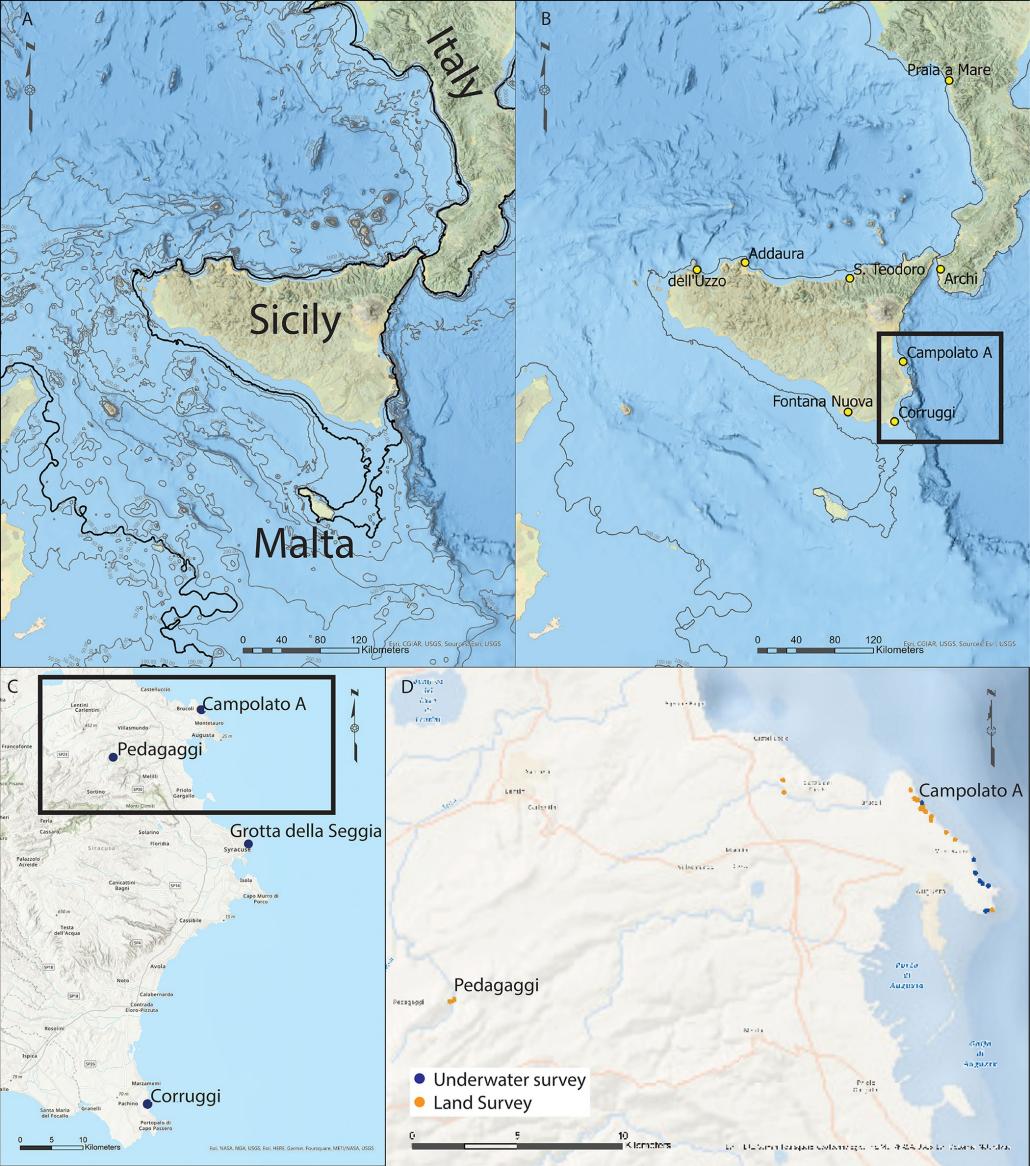
A) Map of Sicily and central Mediterranean showing LGM paleo-coastline in black and detailed bathymetry in gray. B) Same area as a with some sites mentioned in text. Area of interest signaled in black rectangle. C) Close up of study area with position of sites highlighted in text. Survey area signaled in black rectangle. D) Close up of the area surveyed with positions of identified sites.

The apparent late dates for human migration into Sicily indicate either that island occupation is a very late part of human dispersal patterns in Eurasia or that older, formerly coastal sites now lie submerged due to post-Pleistocene sea-level rise. These include the stepped and gradually sloping offshore bathymetry of the eastern portion of Sicily along the Ionian Sea that may preserve now-submerged rockshelters, and topographically flat regions that would have formed a landbridge connecting Sicily to Malta between the LGM and the Holocene [31] (Fig 1A). Even if we accept this late colonization date there are still large gaps in our knowledge of the Sicilian Paleolithic. In fact, the current chronological estimates for the Epigravettian (late Upper Paleolithic) levels are supported by 44 well-published radiometrically dated samples (Table 1) from 12 sites, but these 12 sites are mostly concentrated on the northern shore [3, 9, 13, 15] and the majority (n = 27) of the dates are from just 3 sites along the northern and northwestern shoreline. Moreover, two sites, Acqua Fitusa [8–10] and Grotta Giovanna (the only one in the south of the island [3]), were analyzed before the use of AMS and the dates are therefore considered less reliable. Finally, 20 of the 44 dates are from marine shells and are thus may be less reliable than those from bone or charcoal. Recent genetic data [2] have suggested that Sicilian Epigravettian foragers lived at very low population densities with distinct lineages maintained within the island. Using archaeological data to test these sorts of anthropological questions requires both a larger, and geographically more evenly distributed sample of sites.

The Early Occupation of Sicily (EOS) project was initiated in 2022 in partnership with the Superintendence of Cultural and Natural Heritage of Siracusa and Ragusa, and with the Superintendence of the Sea of Sicily. The project has the aim of extending our understanding of the Paleolithic record of Sicily, with a focus on the southeastern portion of the island (Siracusa and Ragusa Provinces), considering both the terrestrial record and the potentially submerged one (Fig 1). We do this by (a) using unpublished archival data and evidence from avocational archaeologists conducted between the 1870s and the 1990s that remain underreported and absent from most recent syntheses, (b) conducting field survey to relocate known sites and to look for new ones, both on land and underwater, (c), reanalyzing existing museum collections, and (d) outlining new analytical pathways to understand the Sicilian Paleolithic record. By sheer numbers it is clear that although reporting on Sicilian Palaolithic sites goes back to the late 19^th^ century [32], only a very restricted number of sites have been analyzed with modern scientific methods and these are all concentrated in the northern shore of the island. Re-evaluation of sites in other parts of Sicily, and exploration of never before surveyed palaeo-landscapes that are today submerged is crucial not only to create a more complete image of Epigravettian occupation of Sicily, but also to start answering important questions regarding human mobility patterns and human-environmental feedback.

We report here our initial efforts to meet these objectives, including (a) the exact locations for 25 caves and rockshelters found between 1870s and 1990s and then virtually lost, (b) new data resulting from previously unexplored submerged palaeo-coastlines and new work at the Epigravettian sites of Pedgaggi, Campolato Sud A, and Grotta Corruggi, (c) initial results of a reanalysis of the lithic assemblage from Pedagaggi, and (d) developing a protocol for using new and published data to reconstruct patterns of landscape use and social organization among Epigravettian foragers on Sicily.

### Paleolithic Sicily

**Fig 2:**
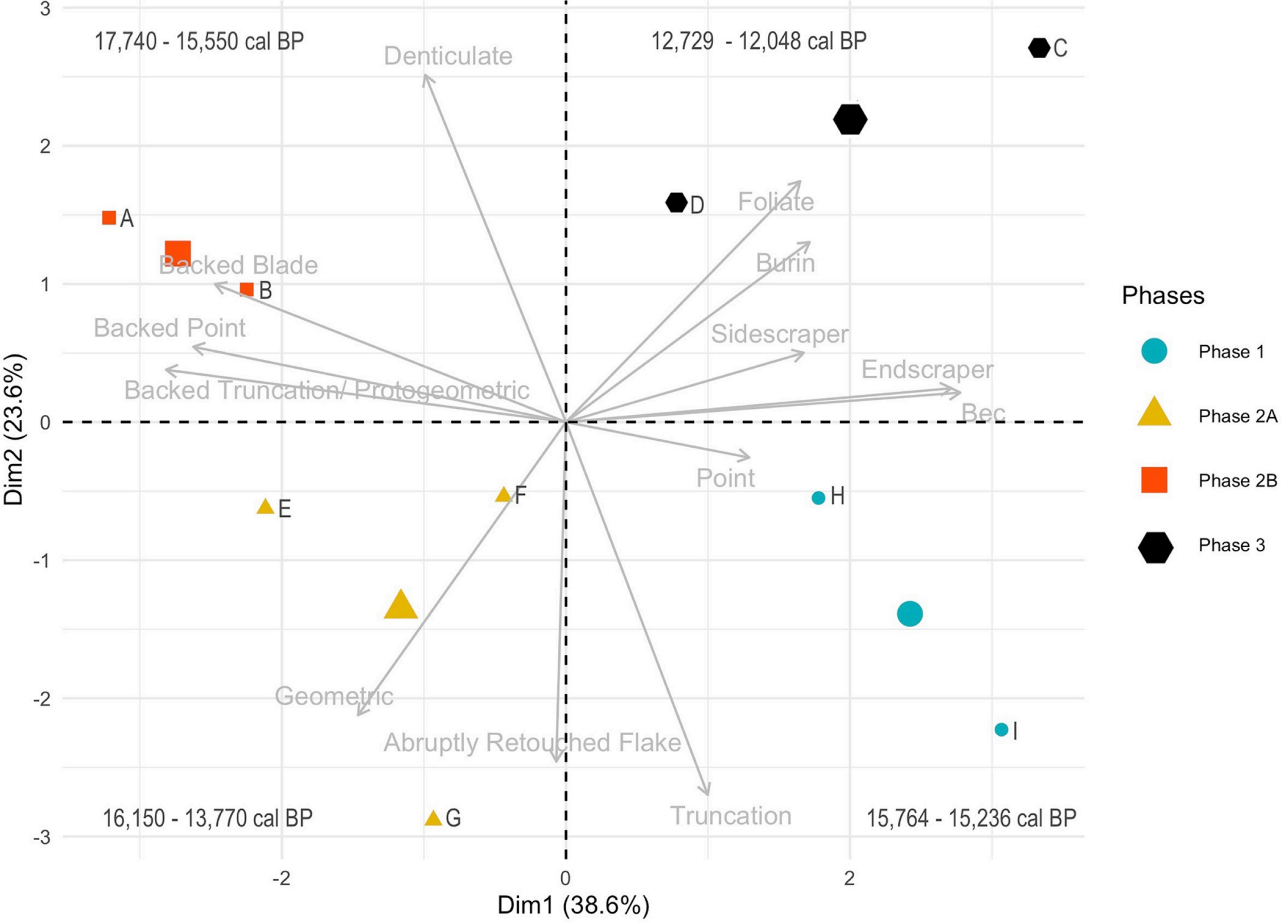
PCA of Epigravettian phases in Sicily as outlined by 12. Chronological phases are divided on a lithic typological frequency basis, with Phase 1 being the oldest. Calibrated radiocarbon dates (collected separately) associated with the sites belonging to each phase are provided, disproving a typological chronology of the Sicilian Epigravettian. A Grotta dell’Acqua Fitusa, superior layer; B Grotta dell’Acqua Fitusa, inferior layer; C Levanzo layer III; D Grotta di San Teodoro, superior layer; E Grotta Giovanna; F Grotta di San Teodoro, inferior layer; G Grotta d’Oriente; H Grotta delle Uccerie, inferior layer; I Grotta delle Uccerie, superior layer. Larger points indicate the midpoint of each phase.

As shown by Di Maida [4] in their recent review of evidence for Palaeolithic occupation of Sicily, pre-LGM migrations on the island, when present, are currently based on surface finds or on collections coming from possibly disturbed palimpsest deposits. The site of Fontana Nuova, in particular, considered Aurignancian (~40–28 ka) by some on lithic technological and typological grounds [cf. 4, 33, 34], now appears to date to the Holocene on the basis of direct radiometric dating. Instead, the earliest evidence is concentrated at ~16–17 ka, consistent with the Late Glacial Epigravettian period seen elsewhere in Italy [35, 36 and summarized in Table 1]. The Epigravettian is defined largely on the basis of lithic technology, which consists primarily of the production of blades and bladelets retouched into a variety of tools including endscrapers and backed points and blades, which Martini et al. [15] divide into a number of ‘phases’ based on the presence, absence, and frequency of different tool types, although the chronological relationship of these phases remains imprecise (Fig 2). Also notable are the multiple ochre-covered burials at San Teodoro and at Grotta d’Oriente [14, 37], and painted or engraved pebbles and rockshelter wall surfaces, as at Cala Genovese [38] and Grotta Addaura [39]. Diets appear to be based on terrestrial food sources until the Holocene [9, 40].

Holistic reconstructions that might provide a good understanding of the paleoenvironmental context for Sicilian Late Pleistocene foragers are largely lacking. Macrobotanical analyses from the Late Pleistocene (but before the LGM) are restricted to charcoal analyses from San Teodoro [41], with pollen also reported from hyena coprolites from the same site [42], sediment samples from the Madonie mountains [43], and cores form Lake Pergusa [21]. Overall, these studies suggest a mosaic of wooded and steppic environments, which are also suggested by the younger post-LGM fossil fauna found at Sicilian Epigravettian archaeological sites (Table 2, Fauna), in particular *C*. *elaphus* and *E*. *hydruntinus* [1, 19]. Stiner and Munro [44] use a ratio of these two taxa as indicators of changing conditions (open to closed habitats) with declining sea level following the LGM at Francthi Cave in Greece, which we report in Table 2. The results suggest substantial variation across the island but are not sufficient for inferring any kind of robust geographic or temporal patterning within Sicily. Detailed zooarchaeological analyses are available only for 11 sites (Table 2), of which 2 only list presence absence of taxa, and thus in general we lack a direct understanding of human interactions with these faunas as an accumulation agent. Isotopic reconstructions from analyses of bones and teeth, while useful, are thus far restricted to the Holocene [e.g., 9, 45].

Three main things are worth noting here in relation to the earliest known human occupation of Sicily: (1) As shown by Antonioli et al. [3] a land bridge connected Sicily to South Italy for at least 1.5 ka during the LGM, between 21.5 and 20 ka. This facilitated European fauna, including *E*. *hydruntinus* and *C*. *elaphus* arriving on the island and eventually replacing the endemic population of mammals [19], (2) The known human occupation postdates the formation of the land bridge, and consists of sites attributed to the Epigravettian, a terminal UP archaeological taxonomic unit also found throughout mainland Italy and adjacent areas, and (3) it is still not clear what is the precise relationship, if any, between the LGM faunal turnover and human arrival on Sicily, although some sort of impact would be predicted by comparison with the coincidence of human arrival and ecological change on other islands within the Mediterranean and elsewhere [46, 47].

## Materials and methods

We concentrated our efforts on the Hyblean Plateau, in the Province of Siracusa (Fig 1). The Hyblean Plateau, which rises to a maximum of 986 m above sea level (asl), consists of Mesozoic and Quaternary carbonate sedimentary successions and marls [48] that lies on the northern portion of the African plate, which collided with the Eurasian plate, which consists of Oligocene calcareous rocks in the west and metamorphic ones in the east [49]. The Hyblean Plateau itself is divided into two tectonic domains by the Tellaro river valley: the Eastern Siracusa one and the western Ragusa one [50]. Because of its tectonic background the Hyblean Plateau forms a quasi-stable coast, having undergone minimum uplift in comparison to the rest of the island, although quantification of the amount of uplift is imprecise because it relies on only two sites [51, 52] The unique tectonic stability of the area facilitates the reconstruction of sea-level changes, simplifying the investigation of paleoshorelines that are now submerged. Therefore, the southeastern corner of Sicily presents a unique opportunity to simultaneously investigate inland and coastal sites during the UP period.

### Site and collections recovery, survey, and description

We used a multi-stage interdisciplinary approach to relocate and assess old collections and sites, and to locate and assess new archaeological sites on land and underwater. Preparatory work included archival research in local town libraries of town and provincial historical bulletins and news articles and self-published articles as far back as the 19^th^ century, many of which are rarely available elsewhere [e.g., 32, 53, 54]. Once we compiled a list of the sites identified in the area of Siracusa we cross-referenced each with the archival lists of the Superintendence for Natural and Cultural Heritage of the Province of Siracusa to find exact locations, official reports and photographs, and physical location of the material recovered. Because photographs or georeferenced coordinates for most sites were rare, and because some sites had multiple designations in sequential reports, we also interviewed local avocational archaeologists, workers for the original excavations, when possible, recreational divers and fishermen. Key collections are maintained at the University of Catania, the Paolo Orsi Museum in Siracusa, and at the Superindence for Cultural and Natural Heritage of Siracusa.

We then surveyed by land and boat aided by historical aerial photographs from the 1966 collection of the Italian Military Geographic Institute and the Geological 1:250.000 map of Sicily [55]; the 1:100.000 Geological map of the Southeast of Sicily [56], the 1:10.000 Regional Technical Map [57] and the 1:50:000 Vulnerability of the Aquifer Map [58]. Our surveys targeted four main areas (Fig 1). Along the coastline, we surveyed (a) ~16 km of continuous coastline from Baia Arcile to Augusta both by land and by boat, with (b) the Santa Panagia area of Siracusa surveyed by boat and underwater diving, and (c) a visit to Grotta Corruggi along the SE shoreline of Sicily (Fig 1). Lastly, in the interior, we surveyed the Serra Paradiso, a valley at the upper reaches of the Gelso River where the site of Pedagaggi is located. Terrestrial survey focused on the investigation of rockshelters and outcrops of Pleistocene terrestrial sediments (i.e., alluvial deposits); we also targeted Marine Isotope Stage 5.e (MIS5.e) (124–119 kya) beaches to quantify tectonic uplift of the modern Siracusa coastline and thus aid in our recovery of submerged deposits, as present rates for the eastern margin of the island are based on only two measured sites [59, 60]. Our marine survey included boat and diving reconnaissance of the same coastline covered during land surveys so to provide new data on partially submerged and submerged caves as well as the presence and conservation of submerged paleosols. To identify submerged anthropogenic sediments, we followed the protocol described in Ogloblin Ramirez et al. [61].

Located sites were mapped (Fig 1 and S1 Table), and in the case of Campolato Sud A, assessed using surface axial seismic tomography to better understand the depth of sediment and buried stratigraphy, therefore the potential for future excavation [62, 63]. Acquisitions and processing were conducted in collaboration with the team of Ceratonia Geophysics. The modeling involved the preliminary inversion of the data through different algorithms, chosen mainly according to the geological characteristics of the investigated area; the final models obtained derive from the inversion according to Occam’s algorithm [64, 65].

Fourier transform infrared spectroscopy (FTIR) was done to describe the mineralogical composition of sediments with the intent of identifying anthropogenic signals such as heated clay [e.g. 66]. Infrared spectra were collected using the KBr method in transmission mode between 4000 and 400cm-1 using a Nicolet iS5 (Thermo Scientific) spectrometer and analyzed with OMNIC 9.3 software. Phytoliths analysis from collected sediments was done following Katz et al. [67]. All sediment was sieved through a 2 mm mesh for the recovery of artifacts.

### Lithic analysis

Our analysis of retouched tools followed the system of Laplace [68–70] because of its wide use in Italian Epigravettian studies, with cores classified using a simple system that takes into consideration the number of surfaces from which flakes were removed, and the orientation of flake removals on those surfaces (single surface unidirectional cores, single surface bidirectional cores, and multi-surface/multi-platform cores). Metric data focused on the weight and maximum linear dimensions of tools, cores, and flakes. These data were then used to assess the degree of winnowing or loss of material using the comparative approach of Schick [71, 72]. The frequency of retouched pieces to cores and flakes per cubic meter of excavated volume was used to assess occupation intensity and landscape patterns following Barton and Riel-Salvatore [73]. Raw material types were assessed visually (aided by a 10x magnification) and the presence of cortex noted, for comparison with known or potential geological sources, especially for chert and quartzite [74–76]. As quartzite is not present on the Hyblean Plateau and must therefore have been transported to sites within it, we use ratios of chert: quartzite retouched pieces to assess material transport and curation.

## Results

### Terrestrial survey along the coast

We recorded ~20 mid-to-Late Pleistocene sites identified and/or excavated between the 1870s and the 1960s along the coastal margin between Augusta and Siracusa, including six partially submerged caves, shown in Fig 1C and S1 Table. While several of the relocated caves still contained sediments, most bore evidence of severe recent human impacts as well as bioturbation. The most drastic of these is Cozzo Telegrafo [77], a cave that was repurposed as a WWII bunker and had all its sediment deposits removed, although we did find several lithic artifacts eroding downslope from the cave openings. Two sites, the Campolato 3 cave [54, 77, 78] and Acquasanta rockshelter [53], contained evidence of almost complete archaeological excavation, with no clear evidence for remaining intact deposit. Campolato 1 cave and the collapsed Campolato rockshelter [77–80] were relocated. Campolato 1 includes a thin (~20–30 cm thick) deposit containing lithic artifacts attributed to the Epigravettian, whereas the rockshelter apparently includes low-density lithic artifacts and fossil fauna from the UP to Roman times and was only minimally tested by Russo [54, 78, 80]. We prioritized two sites that we consider worthy of further study: the rockshelters Campolato Sud A [81] also known as Vallone Amara Nord 3 [54], and Corruggi [82].

**Fig 3:**
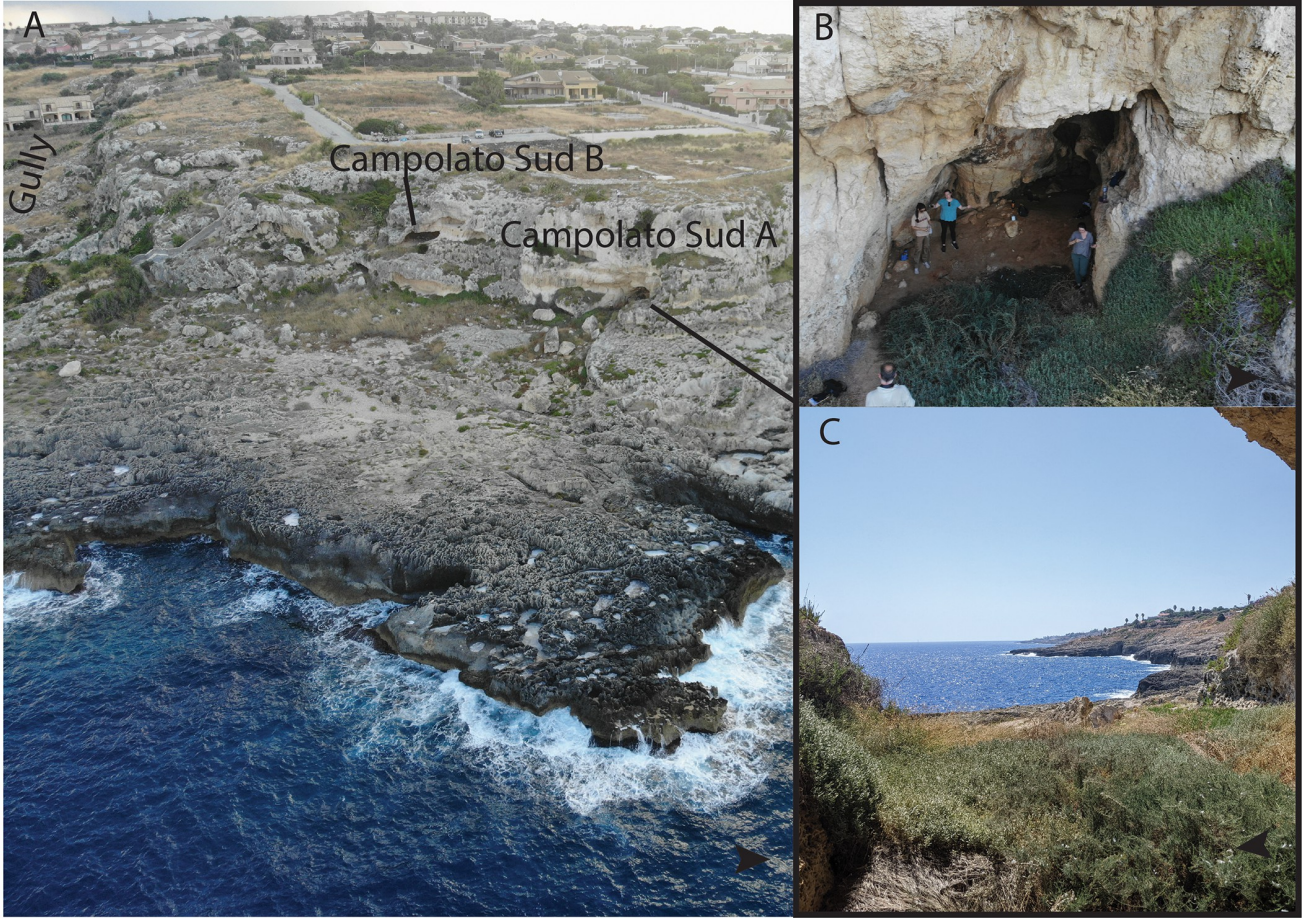
A) aerial view of Campolato site complex; B) view of Campolato Sud A and B note the red sediment in the cave; C) view SE from the cave note the proximity to shore.

**Fig 4:**
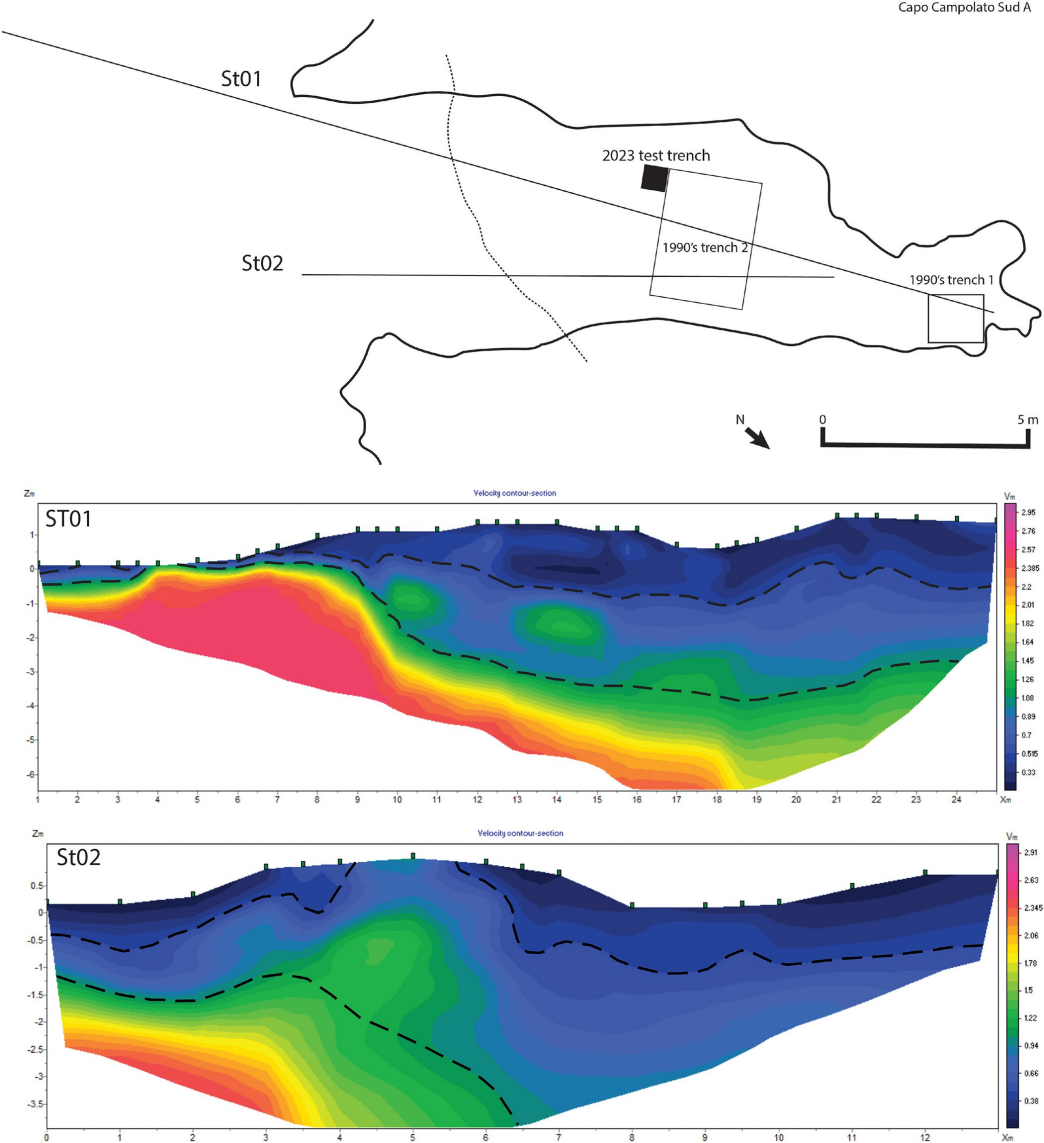
A) Plan view of the Campolato Sud A cave showing the position of 1990s trench and the 2023 test trench. Black lines note the placement of sensors for St01 and St02, dotted line signals the dripline. B) St01 P waves velocity model (in Km/s; RMS error = 4.11%); dashed black lines shows probable seismo-stratigraphic layers. C) St02 P waves velocity model (in Km/s; RMS error = 3.10%); dashed black lines shows probable seismo-stratigraphic layers.

Campolato Sud is a cave complex (Fig 3A) ~30 m from the Ionian Sea near Augusta composed of at least six caves and one rockshelter, part of the Vallone Amara Nord complex of Russo [54]. We focus on Campolato Sud A of Guzzardi [81], also known as Cave 6 of Russo [54]. The rockshelter is small (~17 m deep and 7 m wide) (Figs 2 and 3), with intact red silty deposits visible in the interior of the shelter and in front of it, which contains blocks suggesting recent roof collapse (Fig 3). Russo [54, 78] identifies lithic artifacts he attributes to the Epigravettian eroding from the front of the shelter. The brief report of Guzzardi [81] indicates exploratory excavations at the site to a depth of 1 m that are still visible (Fig 3), with abundant artifacts and faunal assemblages that are rich in deer. We conducted surface axial seismic tomography along two survey lines starting from the outside of the cave and converging at the back wall, one of 25 m with 24 geophones at 1 m interval, and a second of 13 m with 12 geophones (Fig 4A). Analyses of the velocity model allows to distinguish 3 main seismo-stratigraphic layers (Fig 3C and 4B), listed below in stratigraphic order:

• 0.1 < Vp < 0.4/0.6 km/s; low-velocity surface layer, probably consisting of silty sandy matrix soil with pebbles/rock fragments; thickness approx. between 0.0 and 2.0 m;

• 0.4/0.6 < Vp < 1.0/1.2 km/s; intermediate layer, probably consist of blocks in a granular matrix and/or portions of altered/disintegrated rock mass; thickness approx. between 0.3 and 3.5 m;

• Vp > 1.0/1.2 km/s: third layer consisting of rock mass probably in place.

To further assess preservation of surface sediments and possible impact of erosion and bioturbation on the remaining talus sediments we opened a 50x50 cm test trench on the SE corner of the old excavation. We sifted using a 2mm sieve and stopped excavating when we exposed the intact profile and floor of the 1990s excavation at 45 cm below modern floor. No artifacts were recovered from the sieves.

**Fig 5:**
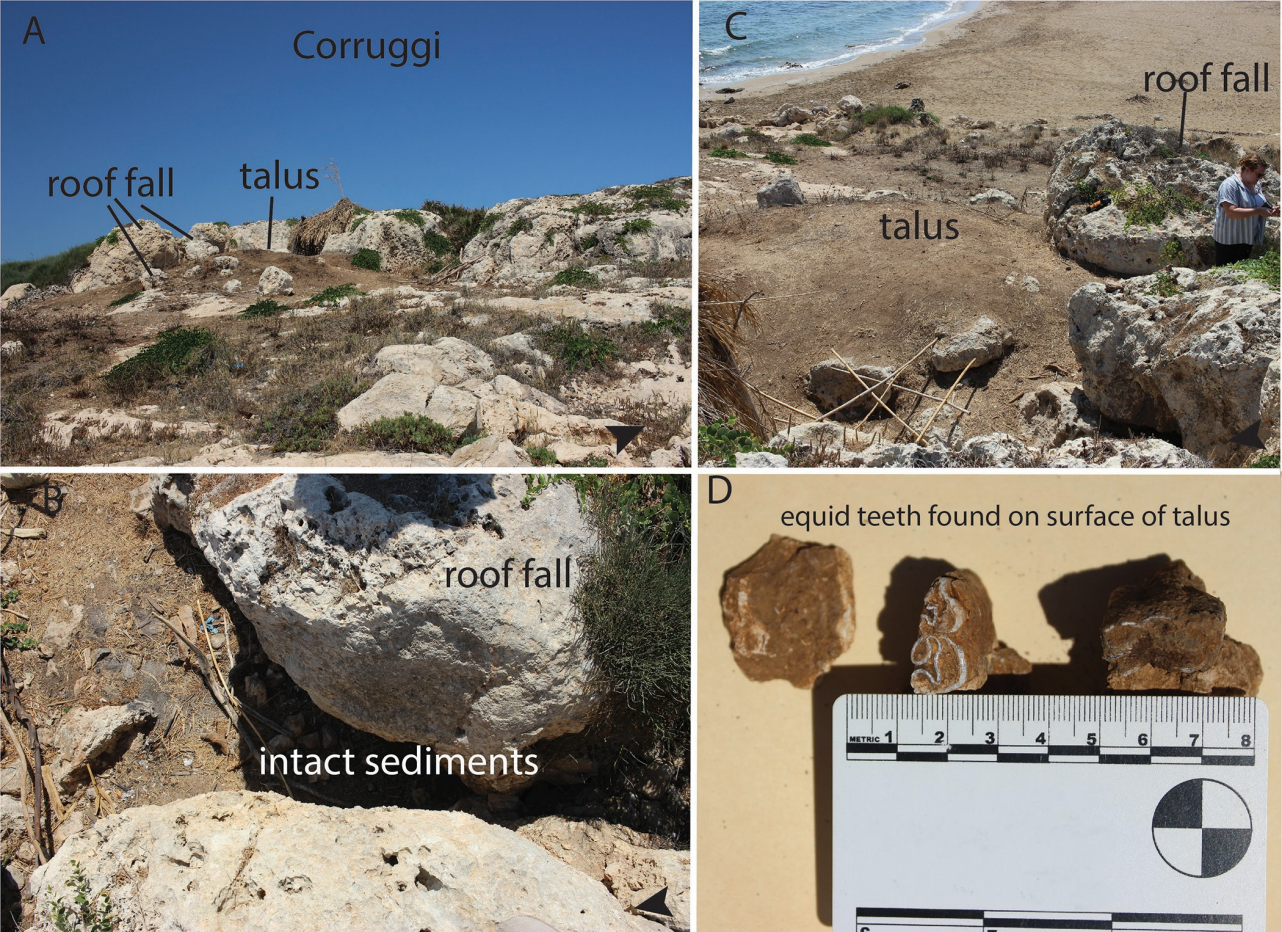
A) view of Corruggi from NE; B) unexcavated area under collapse; C) View SE; D) equid teeth recovered from talus during survey.

The near-shore (<50 m) cave of Corruggi (Fig 5), was discovered and partially excavated by Orsi in 1898 and further excavated by Bernabó Brea in 1945 [82]. The site is located on a limestone terrace in the southernmost part of Sicily’s eastern coastline, facing the LGM land-bridge that would have connected Sicily to Malta. The lithic assemblage from Bernabo Brea’s excavation was studied by Laplace [68], who attributed it to the late Epigravettian, although Martini et al. [15] caution that the Epigravettian layers may have been mixed with overlying ones during excavation. The fauna, studied by Villari [28], does include *E*. *hydruntinus* (Table 2), generally considered extinct by the early Holocene and thus supporting a Pleistocene age for portions of the deposit. In contrast to the other sites we catalogued, the location of this cave was known, however assessment of the remaining sediments had not been carried out. Although most sediments were removed during past excavations, we identified about 2 m^2^ of intact sediment that still remains below the collapsed shelter’s roof (Figs 4B, 4C, and 5A), as well as intact talus deposits (Fig 5C), eroding out of which we observed several chert bladelets and equid teeth identified as *E*. cf. *hydruntinus* (Fig 5D). Per descriptions of past excavations and our survey we believe that the talus of the cave still contains intact sediments that have been minimally impacted by erosion or bioturbation.

### Terrestrial survey of the interior

Survey of the interior focused on the Serra Paradiso, a valley formed near the head of the Gelso stream and site of an important freshwater spring (Fontana del Paradiso), ~20 km inland at an elevation of ~300 m asl (Fig 1C). We focused on this valley because it contained the site of Pedagaggi, an important, if undated Epigravettian assemblage with a detailed typological analysis of the retouched stone tools [83] and a taxonomic assessment of the recovered fossil fauna, which, like Corruggi, included the extinct *E*. *hydruntinus* [27]. However, the site was excavated as a single ~50-cm-thick stratigraphic unit, and concerns remained about the mixing of formerly discrete layers and the age of the site [15]. Our objective was to relocate the site, assess and describe the sedimentary sequence, and to attempt to date the site by radiometric methods. However, the publications provided almost no details on the location of the site (beyond basic dimensions of the shelter), and neither the size nor method of excavation was described. Indeed, the original excavator (Di Geronimo, now >80 years old), had no recollection of the work when interviewed.

**Fig 6:**
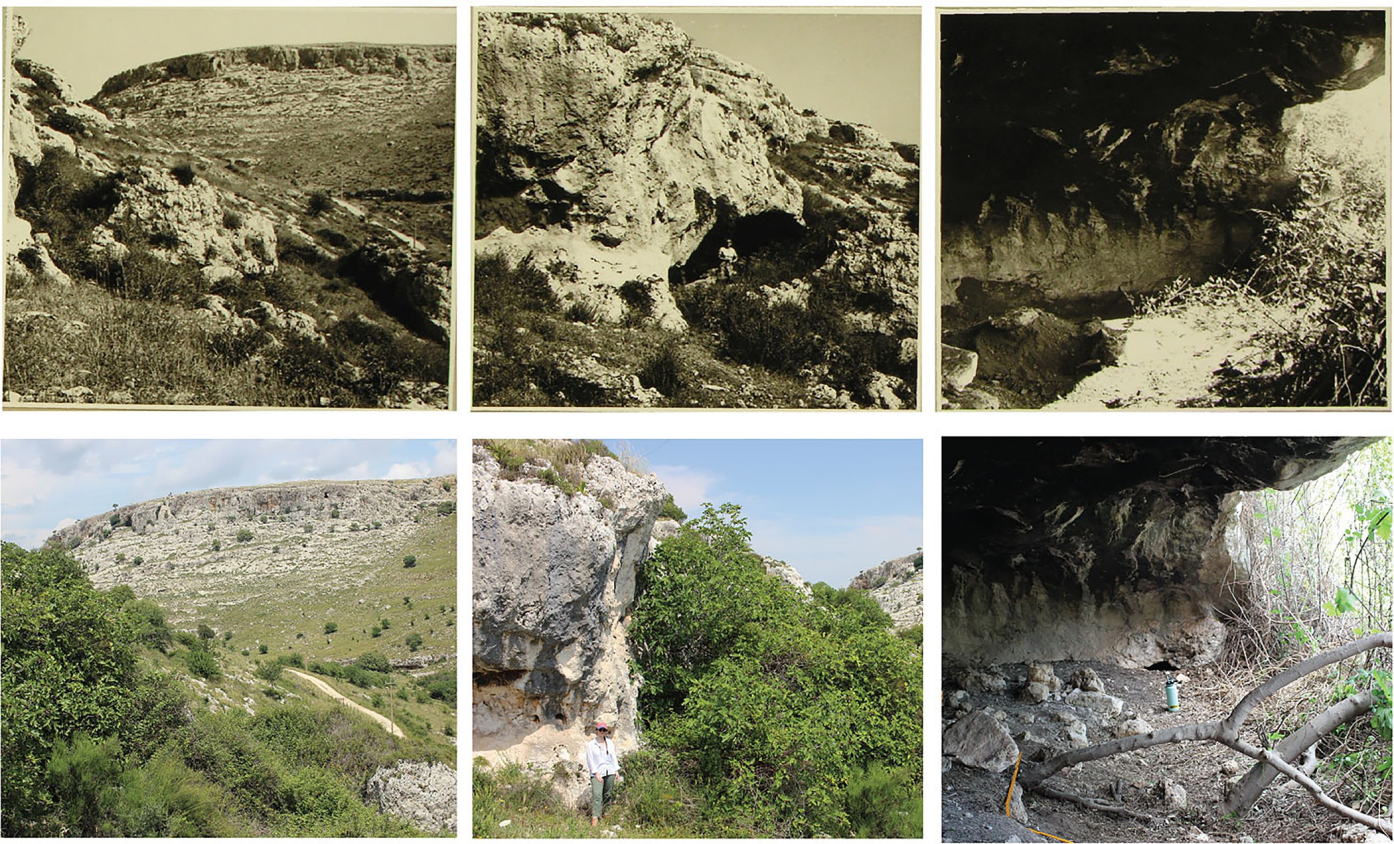
Side by side comparison of 1964 archival photographs of the first discovery of the cave of Pedagaggi and modern pictures showing the cave location today. Note the vegetation that covers the entrance completely rendering the cave almost impenetrable and very difficult to spot from the valley.

Although the Gelso valley has numerous shelters (most with Neolithic to Bronze age deposits) [83–85], we were able to use archival photographs taken during the initial excavation, housed at the Superintendence offices in Siracusa, to relocate the site. Fig 6 shows a side-by-side comparison of the archival photos of Pedagaggi with those taken in 2023. While we were able to locate the site, our own trial excavation confirmed our initial impressions upon arrival that the site had been completely excavated in the past. We were able to estimate the size of their excavation (~12 m^2^). Our sieving of the backdirt and fill using 2 mm mesh resulted in the recovery of multiple fossils and artifacts, including complete flakes <3 cm in maximum dimension. This has important implications for understanding the post-depostional history of the lithic assemblage collected in the 1980s, as detailed below.

Also visible in the Gelso stream is a~20-m-thick sequence of fluvial and lacustrine sediments. The age of these deposits is unknown, but they have the potential to provide a key paleoenvironmental archive for the assemblage.

#### Coastal survey by sea and underwater

**Fig 7:**
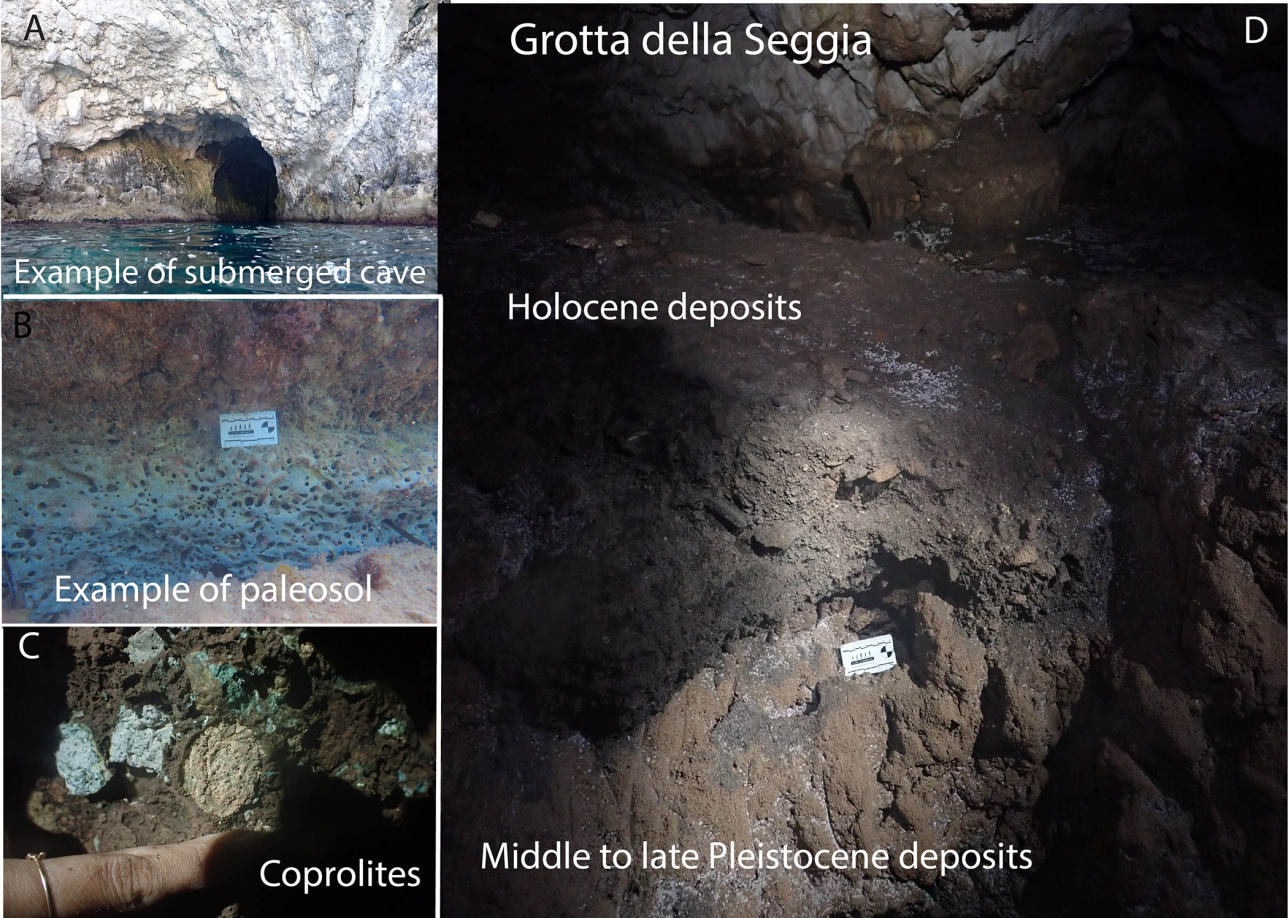
A) submerged cave containing paleosol deposits; B) example of paleosol deposit C) coprolites found in Pleistocene deposits in Grotta della Seggia; D) eroded prolife of sediments in Grotta della Seggia dated to MIS5.e to the Crusader period thanks to fossils and anthropogenic material (e.g. pottery and obsidian blades).

The underwater survey located four submerged caves (one with an intact paleosol), a second intact paleosol, hyena coprolites, and one partially submerged cave, Grotta della Seggia (S2 Table) (Figs 1 and 7). Although no artefacts were recovered from the underwater sites (with the exception of reworked pottery sherds inside one of the submerged caves), we identified the presence of well-preserved palaeosols, which we define as an old soil that has no chemical or physical relation to its modern contextualized environment, that are promising for our future reconstruction of the now submerged landscape, and for the chances of finding submerged Palaeolithic sites. Similar palaeosols containing archaeological remains have been identified in Israel [86] and other regions of the world as United Kingdom [87] or Argentina [88].

Grotta della Seggia (Fig 7) is part of six sites first found >150 years ago [32] and neither explored nor documented since, whose precise locations had been lost. The Grotta della Seggia has three levels indicating multiple stages of formation. The second and middle level contains a terrestrial palimpsest that develops in three chambers and is divided into four sedimentary layers (Fig 7D). The lowermost of these layers contains Middle-Late Pleistocene (pre-LGM) fossils of *Cervus elaphus* and *Palaeoloxodon mnaidriensis* and hyena coprolites, while the uppermost contains Holocene-aged artifacts (e.g., obsidian blades, pottery) attributed to the Neolithic (Stentinello) period, with a bronze coin indicative of even more recent ones. Although lacking Upper Paleolithic material, we used Grotta della Seggia as a model to begin to develop and refine our analytical methods. Loose bulk samples were collected from each layer within the first chamber (S3 Table). The sediment samples were analyzed using FTIR and phytoliths concentrations were quantified.

**Fig 8:**
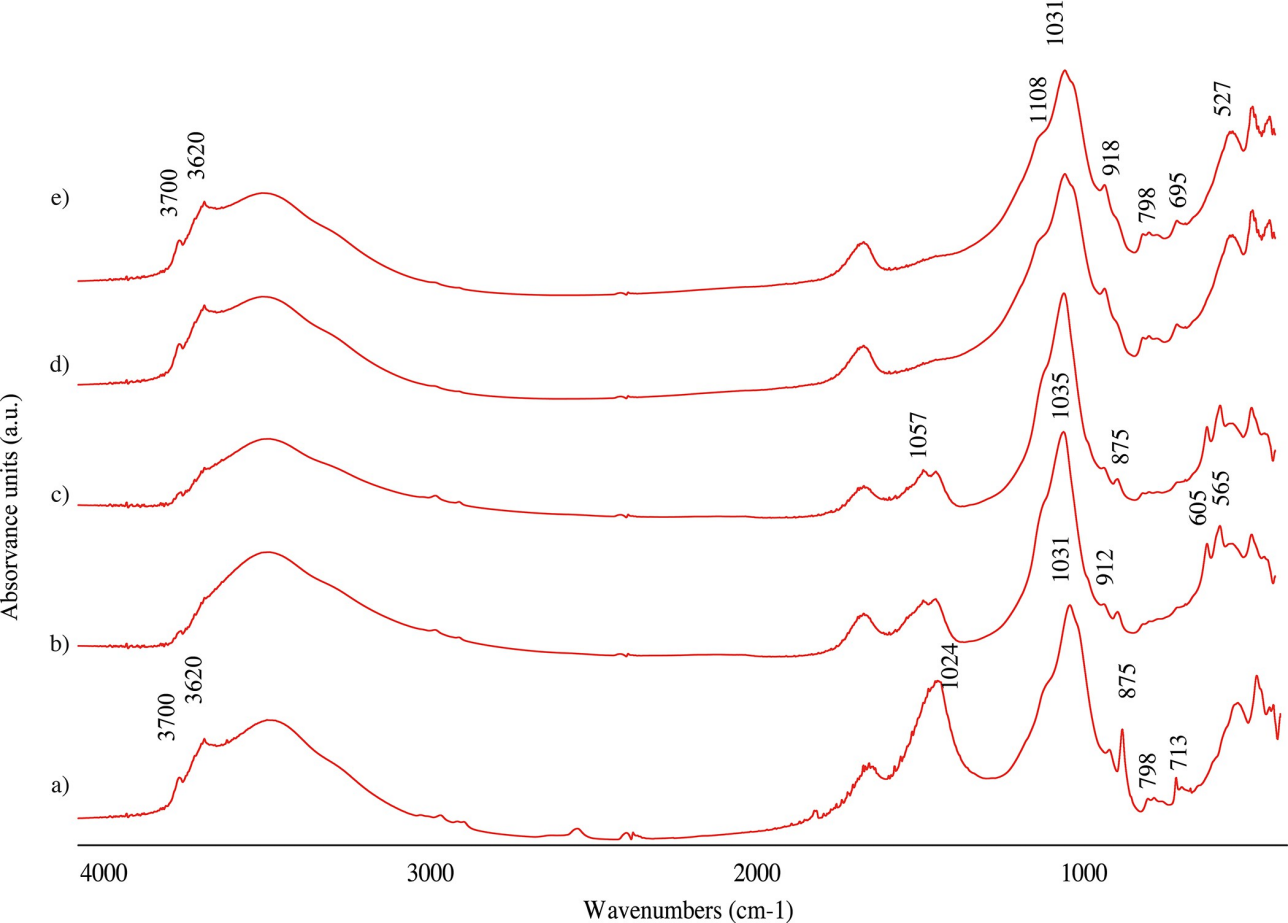
Spectra of layers identified within the first chamber. a) Layer 1. Noted the presence of calcite in contract with Layer 3 (d). b) Contact between Layer 1 and Layer 2. Note the lack of hydroxil peaks around the 3600cm^-1^ and the shift of the main peak of the silicates to a higher number 1035cm^-1^ in comparison to (a). c) Layer 2 has a very similar composition than (b). d and e) Layer 3 and 4 composed of unheated clay and quartz.

**Fig 9:**
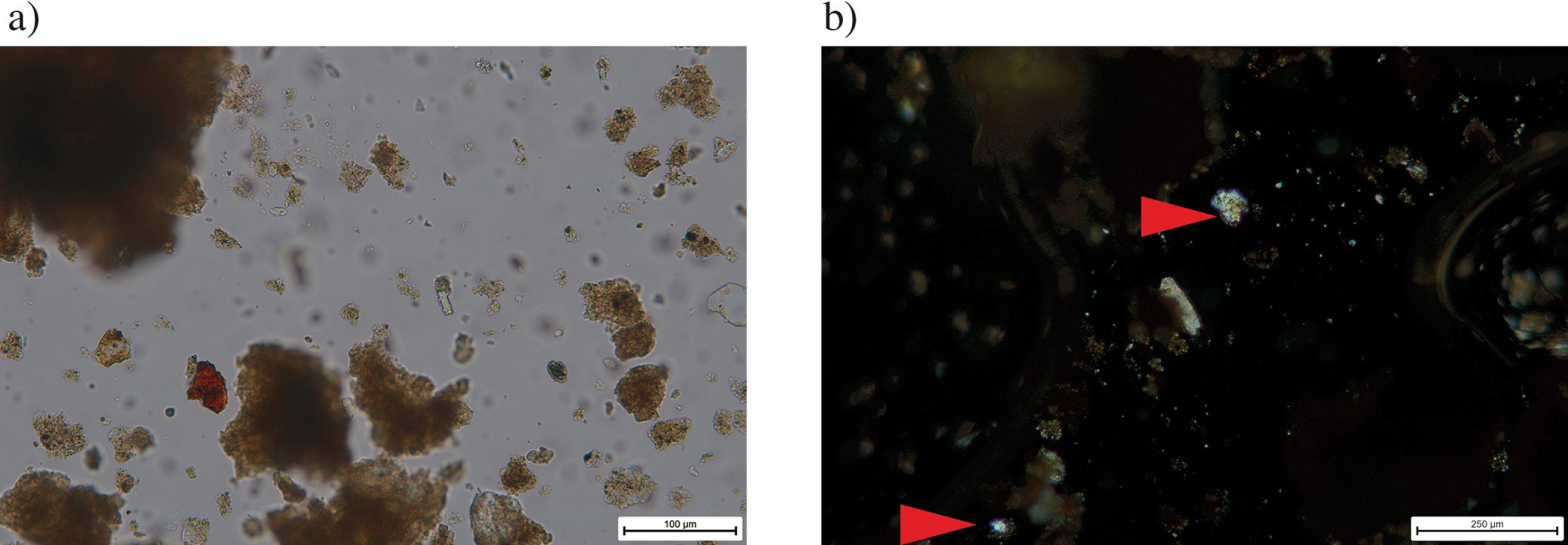
Grain mount observations: a) Long cell phytolith with an unclear morphology probably due to dissolution of opal due to high alkaline conditions [91]. b) Two sub-angular silt grains of quartz found within Layer 1, the lowest level.

The FTIR analyses of the sediment layers show peaks of clays, carbonate hydroxylapatite and quartz. The upper portion of layer 1 in contact with the black crust lacks the presences of peaks at the hydroxyl groups at the 3600cm^-1^ region usually an indication of heat alteration, however, we note the lingering presence of the peak 912cm^-1^ (Fig 8). To assess the possible presence of human controlled fires in these sediments we will complete micromorphological and FTIR microspectroscopy to detect presence/absence of heating above 500°C [89, 90]. Presence of heating right above the mid-Pleistocene faunal remains likely implied human use of the cave. Overall, we note that phytoliths concentrations are too low (between 0.20 and 0.60 million phytoliths per gr of sediment depending on the layer) to accomplish morphological identifications with the protocol adopted. However, the majority of the phytoliths that were observed had poor preservation with indications for dissolution (Fig 9A) [91]. The presence of quartz noted in the FTIR was confirmed preparing grain mounts of the sediment indicating that the layers could be dated using optically stimulated luminescence (OSL) in the future (Fig 8B).

### Shoreline uplift estimates

**Fig 10:**
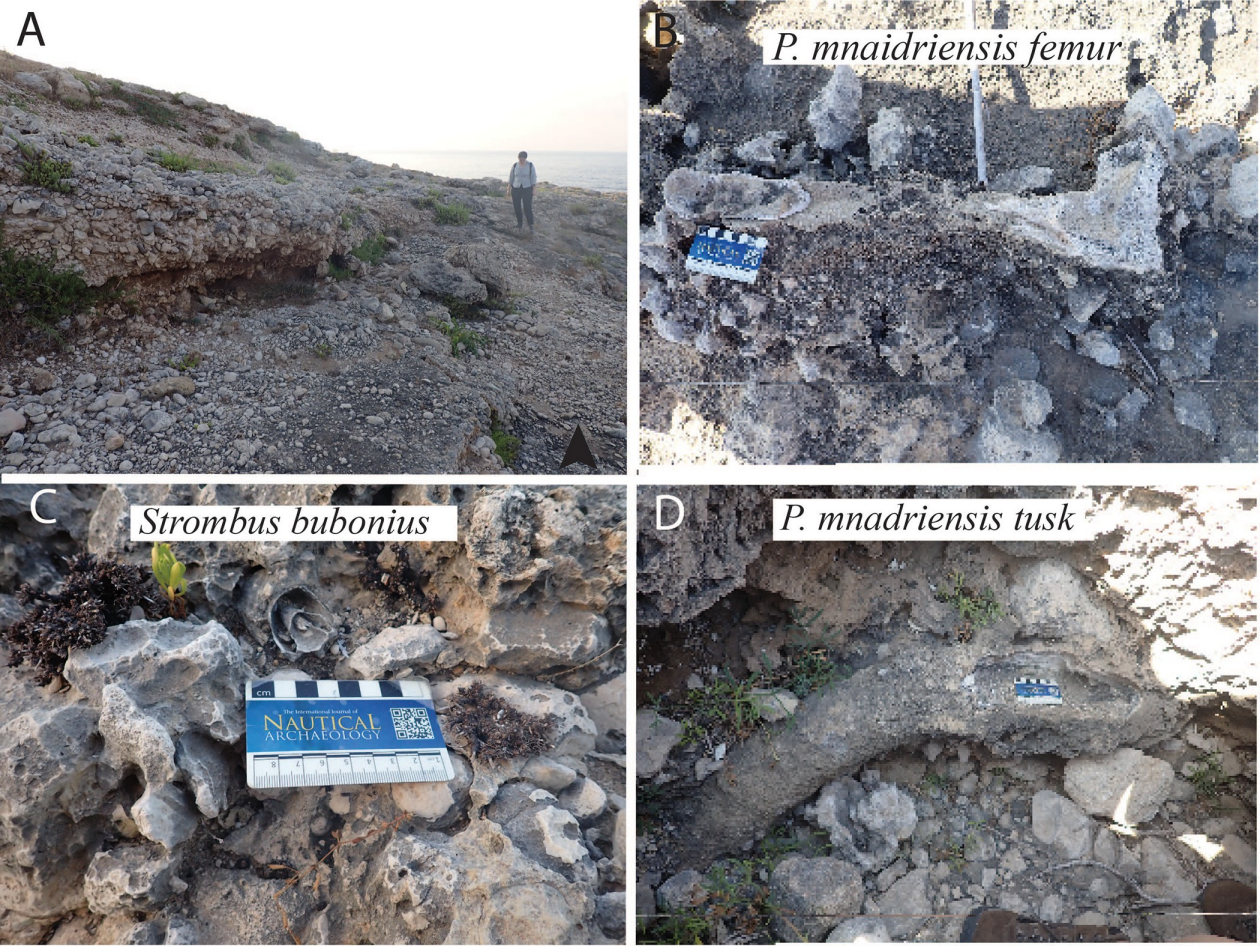
Paleobeaches with associated fossils dated to MIS5.e.

To help quantify uplift in the area we surveyed the coastline for remnants of MIS 5e beaches (Fig 10 and S4 Table). To date them we used the presence of fossil fauna and malacofauna. Starting from the already identified MIS 5e beach [3, 59, 60], we surveyed the coastline of the region north of Augusta and identified three additional beach deposits that can be traced for ~1.5 km containing the Senegalese Guest gastropod *Thetystrombus latus (= Strombus bubonius) and* other index fossils of Sicily. One beach deposit contains elephant remains (femur and tusk). From the size of the femur and the curvature of the tusk we identify this as a possible *P*. *mnaidriensis*, consistent with an MIS 5 age [19]. Our new observations show these beach deposits present at elevations between 6 and 16 meters above sea level (asl) for the area of Augusta (a complete study including postdepositional processes of deposits will follow). Such data are essential for accurate reconstructions of ancient, now submerged shorelines. Our observations reinforce those of Antonioli et al. [3], while highlighting the value of surveying this region in search of coastal Paleolithic sites, as rates and amount of uplift vary across the island. Thus, the SE corner of Sicily is a promising area for the identification of submerged prehistoric sites.

### Reanalysis of museum collections

We reexamined lithic material from the site of Pedagaggi stored at the Paolo Orsi Museum of Siracusa and at the Museum of Paleontology of the University of Catania. The material from the Paolo Orsi Museum consists only of the 11 artifacts collected by M. Mentesana during the initial discovery of the site and described by Bernabo Brea [83]. The collection from the University of Catania, is more significant, and substantially larger (n = 4,442). Di Geronimo et al. [84] provide a comprehensive analysis of the retouched tools following the system of Laplace. We focused on issues of site formation processes, lithic technology, raw material procurement, and site use. Our initial results, reported here, draw largely from published data [83]. A complete reanalysis, including geochemical sourcing, is still ongoing.

**Fig 11:**
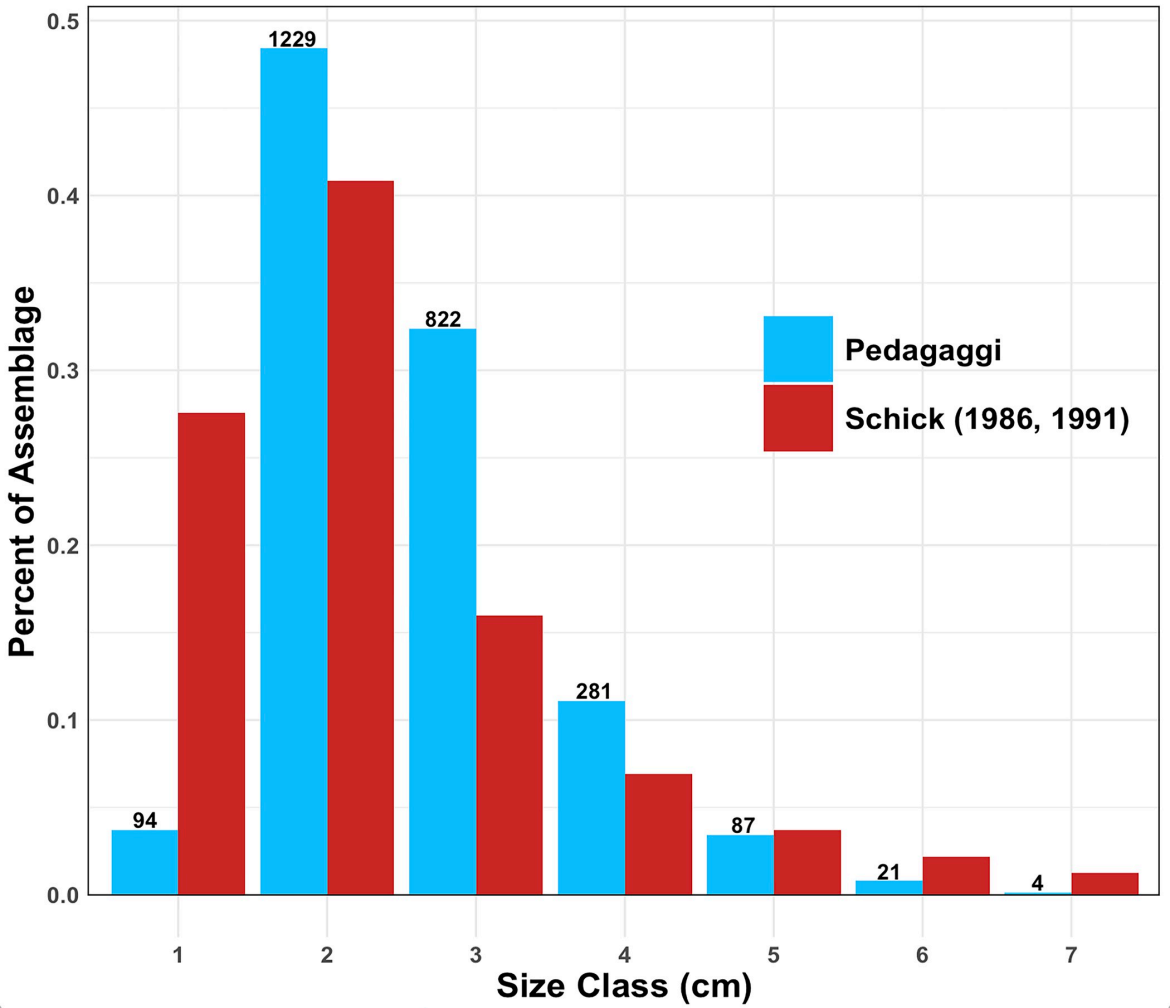
Comparison of the Pedagaggi debitage size distribution to Schick’s [71, 72] experimentally derived assemblage. Pedagaggi artifact counts are listed above each size class.

We examined 2,538 pieces of unretouched flaking debris (a 69% sample of the published total), collecting size data according to the methods of Schick [71, 72], where pieces are binned according to 1-cm size classes. The assemblage is dominated by pieces between 2–3 cm in maximum dimension (Fig 11), consistent with the interpretation that the archaeological collection is the result of on-site artifact production and has been minimally disturbed by post-depositional processes. Experimental core reduction has shown that in fact, pieces < 1 cm in maximum dimension are the most abundant, but the amount recovered archaeologically varies depending on the size of the mesh used. We sieved backdirt and fill from the late 1970s-early 1980s excavation at Pedagaggi using 2 mm mesh, and recovered hundreds of flake fragments as well as complete flakes with dimensions < 1 cm. From this, we infer minimal post-depositional loss of lithic material prior to excavation.

**Fig 12:**
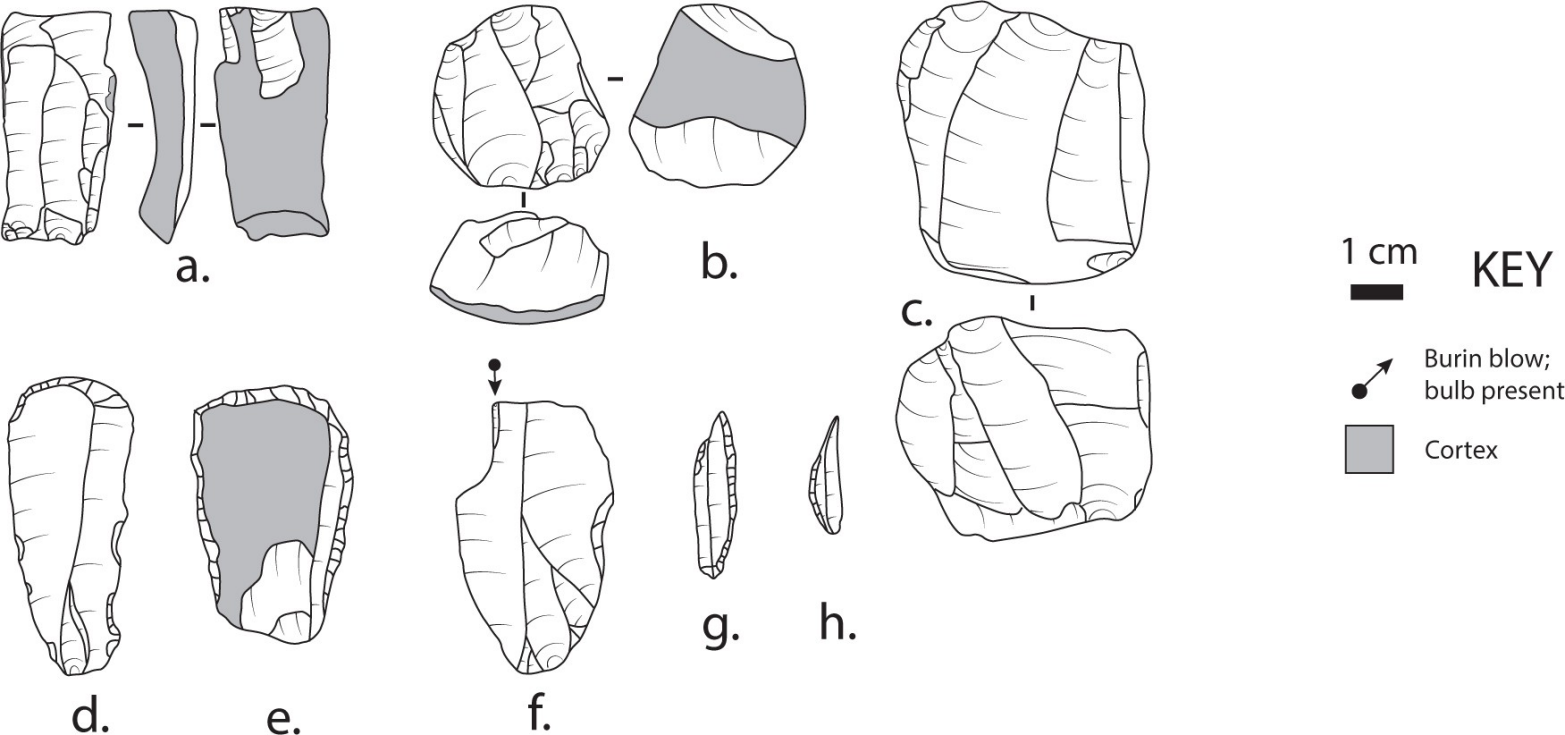
Lithic artifacts from Pedagaggi (all chert). (a) unidirectional core; (b) bidirectional core; (c) multi-surface/multi-platform core; (d-e) endscrapers; (f) burin; (g-h) backed points. Artifacts d-h redrawn from Di Geronimo et al. (1981–1992) [84]. Drawn using a modified version of the STIVA method [93].

Consistent with the application of the Laplace typology, Di Geronimo et al. [84] did not provide details on the cores, which can provide important insights on the technological approaches to flake production. The cores (n = 69) (Fig 12A–12C) are generally small (41.0±9.9 cm in maximum dimension) and consist largely of those flaked unidirectionally or bi-directionally on a single surface (n = 49) for producing laminar blanks (blades or bladelets), and multi-surface/multi-platform and other types (n = 20) for the production of flakes. While classified as tools, some of the burins may have been used primarily for the production of small bladelets (spalls), as suggested by others elsewhere [92].

In terms of raw material, the assemblage is dominated by chert of a variety of different colors, textures, and inclusions (n = 1,625), with rare quartzite (n = 22) and a single piece of vesicular lava. The abundance of cortical pieces is high, with cortex present on >25% of the assemblage. The sources of the raw material are unknown but are almost entirely non-local. The valley itself drains outcrops of basalt and limestone without chert based on inspection of the relevant geological maps and our own extensive targeted and random sampling of cobbles in Gelso stream immediately below the site. These surveys yielded several pieces of very fine-grained basalt; experimental knapping demonstrated the utility of this locally available raw material, yet none of it is present in the excavated assemblage. The source of the chert is unknown, but the nearest known sources are the Leonardo Member of the Ragusa Formation and in the Amerillo Formation, both of which crop out near the town of Monterosso Almo [56], ~20 km from Pedagaggi. Our brief survey of the chert from these sources suggests that it occurs in a range of colors and textures, many similar to those found at Pedagaggi, but definitive geochemical or other means of uniquely identifying particular chert sources in Sicily remain inconclusive [cf. 74, 75] and our own study is just beginning. Importantly, the chert sources near Monterosso Almo are drained by streams that flow *away* from Pedagaggi, on the other side of a major divide within the Hyblean Plateau. This makes it unlikely that closer secondary sources (e.g., as stream clasts) would have been available to the occupants of Pedagaggi.

Quartzite outcrops are not present in the Hyblean Plateau, but secondary sources are likely available as stream clasts in drainage basins at >40 km north of Pedagaggi. Data from Pedagaggi and other sites suggest declining frequency of this material as one moves away from areas where quartzite is present, which tend to be greatest in the northeastern part of the island. Table 3 summarizes our own data from Pedagaggi with published information from other sites. We use a ratio of quartzite to chert tools because data for tools (retouched pieces) are generally available, and we assume a least-effort model where more local raw materials will be predominant at a site. The data suggest a decline in abundance from areas in the northeastern part of Sicily where quartzite is abundant, as at San Teodoro [76] where quartzite tools outnumber chert ones, to lower ratios at sites near the margin of the Hyblean Plateau (Pedagaggi and Capo Campolato 1), to an absence of quartzite at sites far from the margin (Corruggi). These data give some sense of the scale of artifact transport among these Epigravettian sites in southeastern Sicily.

**Table 3:** Published flint and quartzite tool and debitage counts. Sites ordered by geographic location on the island (equally but arbitrarily divided into four quadrants) with references.

Site	Location	Chert Tool Count	Quartzite Tool Count	Chert: Quartzite Tools	Source
**Uccerie, Inferior**	NW	136	0	1: 0	[15]
**Uccerie, Superior**	NW	67	0	1: 0	[15]
**Levanzo, III**	NW	116	0	1: 0	[94]
**Levanzo, II**	NW	69	0	1: 0	[94]
**d’Oriente**	NW	73	0	1: 0	[15]
**San Teodoro, Inferior**	NE	798	458	1: 0.6	[14]
**San Teodoro, Superior**	NE	183	599	1: 3	[14]
**Pierre Sottano, VI**	SE	22	13	1: 0.6	[95]
**Cafici, III**	SE	227	1	1: 0.004	[96]
**Campolato 1**	SE	187	29	1: 0.2	[53]
**Pedagaggi**	SE	572	9	1: 0.02	Data collected by the authors
**San Corrado**	SE	293	0	1: 0	[97]
**Giovanna**	SE	151	0	1: 0	[15]
**Fontana Nuova**	SE	108	0	1: 0	[33]
**Corruggi**	SE	647	0	1: 0	[83]

Lastly, we estimated the artifact volumetric density for Pedagaggi, using the published lithic count data (761 retouched pieces and 3,681 pieces of flakes, flake fragments, and cores) and our estimate of the excavation size (6 m^3^) based on our rediscovery of the site itself. Based on these data, the relative frequency of tools is 20.7 and the artifact volumetric density is 613.5 artifacts/m^3^. Based on comparative data compiled in Heffter [98] for other European Upper Paleolithic sites, the relative frequency of retouch is high and the artifact volumetric density is low. This pattern is consistent with assemblages produced by highly mobile groups [73, 99], and may in part explain the use of stone raw materials from non-local sources.

## Discussion and conclusions

Our current understanding of human dispersal into Sicily relies on a limited number of scientifically analyzed sites concentrated on the northern shore of the island. The paucity of data hinders our reconstructions of timing and means of human arrival and dispersal throughout the island (and to neighboring areas such as Malta) and limits our understanding of Sicilian paleoenvironments and the impacts of humans on them, whether by overhunting, landscape alteration through burning, or other means. The Early Occupation of Sicily (EOS) project was started to increase our understanding of Late Pleistocene human communities and environments on Sicily by focusing on the southeastern portions of the island. The EOS project remains in its initial stages, but aims to increase our understanding of the early human colonization of the Mediterranean islands by enlarging the small sample of well-studied and securely dated sites, including those now underwater that likely included areas preferentially selected for occupation by Late Pleistocene humans.

While we are still in the early stages of the project, we have shown the potential for re-examining historical and archival records in search for sites located, often by avocational archaeologists, which are usually excluded from syntheses of the prehistory of Sicily. Our fieldwork has identified and assessed over 40 sites of interest of which ~17 have been relocated from older identifications, listing for the first time, exact locations for each one. Two of these sites (Campolato Sud A and Corruggi) have the potential of containing Upper Paleolithic (Epigravettian) occupation traces, including fossil fauna, which are targeted for future excavation. We relocated an additional site (Pedagaggi), and while we determined that the site had been completely excavated in the past, were able to estimate the size of the past excavation and to recover important behavioral information by fine-sieving the backdirt and fill.

EOS incorporates a perspective that combines both the land and the sea. We therefore expanded our search to also include submerged paleocoastlines, identifying submerged and partially submerged caves, some of which preserve fossil or artifact-bearing sediments and paleosols, although none yet that match the age of the sites found during our on-land surveys. Our sea survey proves good preservation of submerged and partially submerged Pleistocene sediments and sites in the area between Brucoli and Siracusa proper, promising data for further exploration of the submerged coast, with ongoing development of methods for the analysis and interpretation of the formation and subsequent alteration of sediments in submerged and partially submerged caves. Finally, our analysis of the Epigravettian lithic assemblage from Pedagaggi is the first step in the re-evaluation, curation, and publication of forgotten collections. Our results suggest that the majority of the lithic assemblage is preserved, that core reduction was oriented toward the production of blades and bladelets and was likely produced by highly mobile foragers relying on non-local sources of stone. Building on these data requires an expanded data set for comparison, something we look forward to doing in the near future.

## Supporting information

S1 Table List of land sites identified during survey. (DOCX)

S2 Table List of underwater caves and clay deposits. Submerged caves and paleosols were found during our boat and underwater surveys.
(DOCX)

S3 Table List of sediment samples from Grotta della Seggia. Mineralogical composition was determined by FTIR spectroscopy: Cl = Clay, (?) = Possible altered clay, (u/a) = unaltered by heat, Qz = quartz, Ca = Calcite, CHAP = carbonate hydroxylapatite. Phytoliths quantification are expressed in million per gram of sediment.
(DOCX)

S4 Table List of beach depositis used to quantify uplift since MIS5e. (DOCX)

## References

[1] Cherry JF, Leppard TP. Patterning and its causation in the pre-Neolithic colonization of the Mediterranean islands (Late Pleistocene to Early Holocene). The Journal of Island and Coastal Archaeology. 2018 Apr 3;13(2):191–205.

[2] Slimak L. The three waves: Rethinking the structure of the first Upper Paleolithic in Western Eurasia. Plos one. 2023 May 3;18(5): e0277444. doi: 10.1371/journal.pone.0277444 37134082 PMC10155996

[3] Antonioli F, Lo Presti V, Morticelli MG, Bonfiglio L, Mannino MA, Palombo MR, et al. Timing of the emergence of the Europe–Sicily bridge (40–17 cal ka BP) and its implications for the spread of modern humans. Geological Society, London, Special Publications. 2016;411(1):111–44.

[4] Di Maida G. The earliest human occupation of Sicily: A review. The Journal of Island and Coastal Archaeology. 2022 Jul 22;17(3):402–19.

[5] Fu Q., Li H., Moorjani P., Jay F., Slepchenko S.M., Bondarev A.A., et al. Genome sequence of a 45,000-year-old modern human from western Siberia. Nature, 2014. 514: 445–449. doi: 10.1038/nature13810 25341783 PMC4753769

[6] Bird MI, Beaman RJ, Condie SA, Cooper A, Ulm S, Veth P. Palaeogeography and voyage modeling indicates early human colonization of Australia was likely from Timor-Roti. Quaternary Science Reviews. 2018 Jul 1;191:431–9.

[7] Crema ER, Bevan A. Inference from large sets of radiocarbon dates: software and methods. Radiocarbon. 2021 Feb;63(1):23–39.

[8] Azzi CM, Bigliocca L, Piovan E. Florence radiocarbon dates I. Radiocarbon. 1973;15(3):479–87.

[9] Mannino MA, Thomas KD. New radiocarbon dates for hunter-gatherers and early farmers in Sicily. Accordia Research Papers. 2007;10:13–34.

[10] Caramia F. L’industria litica epigravettiana di Grotta dell’Acqua Fitusa (Agrigento): nuove acquisizioni tecno-tipologiche. L’industria litica epigravettiana di Grotta dell’Acqua Fitusa (Agrigento): nuove acquisizioni tecno-tipologiche. 2005:213–34.

[11] Nicoletti F., & Tusa S. Nuove acquisizioni scientifiche sul Riparo del Castello di Termini Imerese (Palermo) nel quadro della preistoria siciliana tra la fine del Pleistocene e gli inizi dell’Olocene. Nuove acquisizioni scientifiche sul Riparo del Castello di Termini Imerese (Palermo) nel quadro della preistoria siciliana tra la fine del Pleistocene e gli inizi dell’Olocene, 2012 303–318.

[12] Catalano G, Vetro DL, Fabbri PF, Mallick S, Reich D, Rohland N, et al. Late Upper Palaeolithic hunter-gatherers in the Central Mediterranean: New archaeological and genetic data from the Late Epigravettian burial Oriente C (Favignana, Sicily). Quaternary International. 2020 Jan 30;537:24–32. 10.1016/j.quaint.2020.01.025 PMC11580030 39574513

[13] Di Maida G, Mannino MA, Zilhão J, Hoffmann DL, García-Diez M, Pastoors A, et al. Radiocarbon and U-series age constraints for the Lateglacial rock art of Sicily. Quaternary Science Reviews. 2020 Oct 1;245:106524.

[14] Garilli V, Vita G, Mulone A, Bonfiglio L, Sineo L. From sepulchre to butchery-cooking: Facies analysis, taphonomy and stratigraphy of the Upper Palaeolithic post burial layer from the San Teodoro Cave (NE Sicily) reveal change in the use of the site. Journal of Archaeological Science: Reports. 2020 Apr 1;30:102191.

[15] Martini F., Vetro D. L., Colonese A. C., De Curtis O., Di Giuseppe Z., Locatelli E., et al. L’Epigravettiano finale in Sicilia. In L’Italia tra 15.000 e 10.000 anni fa. Cosmopolistismo e regionalita nel tardoglaciale. Martini F, editor. L’Italia tra 15.000 e 10.000 anni fa: cosmopolitismo e regionalità nel tardoglaciale: atti della tavola rotonda (Firenze, 18 novembre 2005). Museo Fiorentino di Preistoria" Paolo Graziosi"; 2007 209–254).

[16] Leighton R. Sicily before history: an archaeological survey from the Palaeolithic to the Iron Age. Cornell University Press; 1999.

[17] D’Amore G, Di Marco S, Tartarelli G, Bigazzi R, Sineo L. Late Pleistocene human evolution in Sicily: comparative morphometric analysis of Grotta di San Teodoro craniofacial remains. Journal of Human Evolution. 2009 Jun 1;56(6):537–50. doi: 10.1016/j.jhevol.2009.02.002 19446307

[18] Posth C, Yu H, Ghalichi A, Rougier H, Crevecoeur I, Huang Y, et al. Palaeogenomics of Upper Palaeolithic to Neolithic European hunter-gatherers. Nature. 2023 Mar 2;615(7950):117–26. doi: 10.1038/s41586-023-05726-0 36859578 PMC9977688

[19] Bonfiglio L., Insacco G, Marra AC, Masini F. Large and small mammals, amphibians and reptiles from a new late Pleistocene fissure filling deposit of the Hyblean Plateau (South Eastern Sicily). Bollettino Societa Paleontologica Italiana. 1997;36:97–122.

[20] Masini F., Petruso D., Bonfiglio L., Mangano G. Origination and extinction patterns of Mammals in three Central Western Mediterranean Islands in the Late Miocene to Quaternary”. Quat. Intern. 2008,182, pp. 63–79

[21] Sadori L, Zanchetta G, Giardini M. Last Glacial to Holocene palaeoenvironmental evolution at Lago di Pergusa (Sicily, Southern Italy) as inferred by pollen, microcharcoal, and stable isotopes. Quaternary International. 2008 Apr 1;181(1):4–14.

[22] Magny M, Vannière B, Calo C, Millet L, Leroux A, Peyron O, et al. Holocene hydrological changes in south-western Mediterranean as recorded by lake-level fluctuations at Lago Preola, a coastal lake in southern Sicily, Italy. Quaternary Science Reviews. 2011 Sep 1;30(19–20):2459–75.

[23] Tagliacozzo Antonio. Archeozoologia della Grotta dell’Uzzo, Sicilia: Da un’economia di pesca ed allevamento. Bullettino di paletnologia italiana 1993; 84.

[24] Mannino MA, Talamo S, Tagliacozzo A, Fiore I, Nehlich O, Piperno M, et al. Climate-driven environmental changes around 8,200 years ago favoured increases in cetacean strandings and Mediterranean hunter-gatherers exploited them. Scientific Reports. 2015 Nov 17;5(1):1–2. 10.1038/srep16288 PMC4648091 26573384

[25] Vigliardi Alda. Gli Strati Paleo-Mesolitici Della Grotta Di Levanzo. *Rivista Di Scienze Preistoriche* 1982. 37: 79–13.

[26] Regàlia E. 1907. Sulla Fauna della « Grotta del Castello » di Termini Imerese (Palermo). Archivio per l’antropologia e la etnologia 1907. 37(3): 337–373.

[27] Gliozzi E, Kostakis T, I vertebrati fossili del giacimento epigravettiano finale di Pedagaggi (Siracusa, Sicilia orientale), Il Naturalista siciliano, Palermo, 1986 10, 35–42

[28] Villari P. Le faune della tarda preistoria della Sicilia orientale. Ente fauna siciliana; 1995.

[29] Brea LB. Yacimientos paleolíticos del sudeste de Sicilia. Empúries: revista de món clàssic i antiguitat tardana. 1950:115–43

[30] Cardini L. Rinvenimenti Paleolitici nella Grotta Giovanna (Siracusa) Atti XIII Riunione Scientifica I.I.P.P. Firenze, 1971:29–35

[31] Foglini F, Prampolini M, Micallef A, Angeletti L, Vandelli V, Deidun A, et al. Late Quaternary coastal landscape morphology and evolution of the Maltese Islands (Mediterranean Sea) reconstructed from high-resolution seafloor data. Special Publications. 2016;411(1):77–95.

[32] Von Andrian F. 1878. Prähistorische Studien aus Sicilien in Zeitschrift fúr Ethologie Wiegandt, Hempel & Parey; 1878 1:89

[33] Chilardi S, Frayer DW, Gioia P, Macchiarelli R, Mussi M. Fontana Nuova di Ragusa (Sicily, Italy): southernmost Aurignacian site in Europe. Antiquity. 1996 Sep;70(269):553–63.

[34] Pianese S. P. Rassegna storica delle ricerche sul Paleolitico in Sicilia. Quaternaria, 1968 10, 213–250.

[35] Ruiz-Redondo A, Vukosavljević N, Tomasso A, Peresani M, Davies W, Vander Linden M. Mid and Late Upper Palaeolithic in the Adriatic Basin: Chronology, transitions and human adaptations to a changing landscape. Quaternary Science Reviews. 2022 Jan 15;276:107319.

[36] Naudinot N, Tomasso A, Tozzi C, Peresani M. Changes in mobility patterns as a factor of 14C date density variation in the Late Epigravettian of Northern Italy and Southeastern France. Journal of Archaeological Science. 2014 Dec 1;52:578–90.

[37] Di Salvo R, Mannino G, Mannino MA, Schimmenti V, Sineo L. Le sepolture della Grotta d’Oriente (Favignana). Le sepolture della Grotta d’Oriente (Favignana). 2012:341–51.

[38] Cultraro M. Grotta di Cala Genovese a Levanzo. Pasquale A., Tarantini M., a cura di, Segni dalla preistoria: siti. 2018.

[39] Mannino G. I graffiti parietali preistorici della Grotta Addaura: la scoperta e nuove acquisizioni. I graffiti parietali preistorici della Grotta Addaura: la scoperta e nuove acquisizioni. 2012:415–22.

[40] Yu H, van de Loosdrecht MS, Mannino MA, Talamo S, Rohrlach AB, Childebayeva A, et al. Genomic and dietary discontinuities during the Mesolithic and Neolithic in Sicily. Iscience. 2022 May 20;25(5). doi: 10.1016/j.isci.2022.104244 35494246 PMC9051636

[41] Lona F. Ricerche sulla flora quaternaria italiana. I carboni dei focolari paleolitici della Grotta di S. Teodoro (Messina). Rivista di Scienze Preistoriche. 1949;4:187–93.

[42] Yll R, Carrión JS, Marra AC, Bonfiglio L. Vegetation reconstruction on the basis of pollen in Late Pleistocene hyena coprolites from San Teodoro Cave (Sicily, Italy). Palaeogeography, Palaeoclimatology, Palaeoecology. 2006 Jul 21;237(1):32–9.

[43] Marchetti D.B., Accorsi C.A., Arobba D., Mazzanti M.B., Bertolani M., Biondi E., et al. Recherches géobotaniques sùr les Monts Madonie (Sicile du nord). Webbia, 1984 38(1), pp.329–348.

[44] Stiner MC, Munro ND. On the evolution of diet and landscape during the Upper Paleolithic through Mesolithic at Franchthi Cave (Peloponnese, Greece). Journal of Human Evolution. 2011 May 1;60(5):618–36. doi: 10.1016/j.jhevol.2010.12.005 21371735

[45] Di Maida G, Mannino MA, Krause-Kyora B, Jensen TZ, Talamo S. Radiocarbon dating and isotope analysis on the purported Aurignacian skeletal remains from Fontana Nuova (Ragusa, Italy). Plos one. 2019 Mar 20;14(3): e0213173. doi: 10.1371/journal.pone.0213173 30893326 PMC6426221

[46] Alcover JA, Seguí B, Bover P. Extinctions and local disappearances of vertebrates in the western Mediterranean islands. Extinctions in near time: causes, contexts, and consequences. 1999:165–88.

[47] Blondel J. On humans and wildlife in Mediterranean islands. Journal of Biogeography. 2008 Mar;35(3):509–18.

[48] Lentini F, Carbone S, Catalano S. Main structural domains of the central Mediterranean region and their Neogene tectonic evolution. Bollettino di Geofisica Teorica ed Applicata. 1994;36(141–44):103–25.

[49] Lentini F, Carbone S, Barreca G. The Tyrrhenian stage geodinamic evolution of Apenninic-Maghrebian orogen (Southern Apennines and Sicily). InEGU General Assembly Conference Abstracts 2009 Apr (p. 10424).

[50] Ghisetti F, Vezzani L. The structural features of the Iblean Plateau and of the Mount Judica area (southeastern Sicily); a microtectonic contribution to the deformational history of the Calabrian Arc. Bollettino della Società Geologica Italiana. 1980 Jan 1;99(1–2):57–102.

[51] Antonioli F, Kershaw S, Renda P, Rust D, Belluomini G, Cerasoli M, et al. Elevation of the last interglacial highstand in Sicily (Italy): a benchmark of coastal tectonics. Quaternary International. 2006 Mar 1;145:3–18.

[52] Bianca M, Monaco C, Tortorici L, Cernobori L. Quaternary normal faulting in southeastern Sicily (Italy): a seismic source for the 1693 large earthquake. Geophysical Journal International. 1999 Nov;139(2):370–94.

[53] Mentesana M., Su una Caverna Preistorica di Monte Amara, Notiziario Storico di Augusta, 2, 06, 1968

[54] Russo I., Il Paleolitico Superiore e la Tradizione Paleolitica sul Tauro di Augusta, Quaderni di Archeologia Preistorica, Edizioni del Medirraneo, 2002. 6.

[55] Lentini F., Carbone S., Carta Geologica Della Sicilia, Dipartimento di Scienze Geologiche, Università di Catania; 2009

[56] Lentini F. Carta Geologica della sicilia sud-orientale. Catania: Università di Catania, Istituto di Scienze della Terra; 1987.

[57] Cirone S., Carta Technica Regionale Sezione N. 641110 Brucoli, Regione Siciliana Assessorato Regionale del Territorio e dell’Ambiente Dipartimento dell’Urbanistica, 2014

[58] Aureli A. Carta della vulnerabilità delle Falde Idriche: Settore Sud-Orientale ibleo (Sicilia S.E.). S.l.: s.n.; 1990.

[59] Di Grande A, Scamarda G. Segnalazione di livelli a Strombus bubonius Lamarck nei dintorni di Augusta (Siracusa). Tipografia Monforte; 1973.

[60] Di Grande A, Neri M. Tirreniano a Strombus bubonius a M. Tauro (Augusta-Siracusa). Rendiconti SocietaGeologica Italiana. 1988;11:57–8.

[61] Ogloblin Ramirez I, Galili E, Shahack-Gross R. Locating submerged prehistoric settlements: A new underwater survey method using water-jet coring and micro-geoarchaeological techniques. Journal of Archaeological Science. 2021 Nov 1;135:105480.

[62] Corradini E, Eriksen BV, Mortensen MF, Nielsen MK, Thorwart M, Krüger S, et al. Investigating lake sediments and peat deposits with geophysical methods-A case study from a kettle hole at the Late Palaeolithic site of Tyrsted, Denmark. Quaternary International. 2020 Aug 30;558:89–106.

[63] Sarris A, Kalayci T, Moffat I, Manataki M. An introduction to geophysical and geochemical methods in digital geoarchaeology. Digital Geoarchaeology: New Techniques for Interdisciplinary Human-Environmental Research. 2018 215–36.

[64] Constable SC, Parker RL, Constable CG. Occam’s inversion: A practical algorithm for generating smooth models from electromagnetic sounding data. Geophysics. 1987 Mar;52(3):289–300.

[65] deGroot-Hedlin C, Constable S. Occam’s inversion to generate smooth, two-dimensional models from magnetotelluric data. Geophysics. 1990 Dec;55(12):1613–24.

[66] Berna F, Behar A, Shahack-Gross R, Berg J, Boaretto E, Gilboa A, et al. Sediments exposed to high temperatures: reconstructing pyrotechnological processes in Late Bronze and Iron Age Strata at Tel Dor (Israel). Journal of Archaeological Science. 2007 Mar 1;34(3):358–73.

[67] Katz O, Cabanes D, Weiner S, Maeir AM, Boaretto E, Shahack-Gross R. Rapid phytolith extraction for analysis of phytolith concentrations and assemblages during an excavation: an application at Tell es-Safi/Gath, Israel. Journal of Archaeological Science. 2010 Jul 1;37(7):1557–63

[68] Laplace G. Recherches sur l’origine et l’évolution des complexes leptolithiques. Persée-Portail des revues scientifiques en SHS; 1966.

[69] Laplace G., Recherches de Typologie analytique. Origini, 1968 2:7–63.

[70] Laplace G., Liste typologique 1972. Cahiers de typologie analytique, 1972 9–27.

[71] Schick KD. Stone Age sites in the making: experiments in the formation and transformation of archaeological occurrences. BAR Publishing; 1986.

[72] Schick K., On making behavioral inferences from early archaeological sites. In (Clark J.D., ed.): Cultural Beginnings: Approaches to Understanding Early Hominid Lifeways in the African Savanna. Bonn: Rudolph Hablelt GMBH. 1991 79–107.

[73] Barton C.M., Riel-Salvatore J. The formation of lithic assemblages. Journal of Archaeological Science, 2014 46:334–352.

[74] Fitula M., Flints from Akrai. In: Chowaniec R., (Eds.), Unveiling the past of an ancient town Akrai/Acrae in Southeastern Sicily. Warsaw: University of Warsaw. 2015

[75] Chatzimpaloglou P. A geoarchaeological methodology for sourcing chert artefacts in the Mediterranean region: A case study from Neolithic Skorba on Malta. Geoarchaeology. 2020 Nov;35(6):897–920.

[76] Vita G, Forgia V, Vraca MP, Calabrese N, Divita D, Sineo L. Petrographic characterization of quartzite tools from the Palaeolithic site of San Teodoro cave (Sicily): Study on the provenance of lithic raw materials. Journal of Archaeological Science: Reports. 2022 Oct 1;45:103593.

[77] Russo I; Gianino P. L’età della pietra nel territorio di Augusta. Notizaiaro Storico di Augusta. 1987 15: 5–25

[78] Russo I. Campolato: Una stazione paleolitica. Notizaiaro Storico di Augusta. 1981 10: 23–27.

[79] Lanteri R., Augusta e il suo territorio: elementi per una carta archeologica, Catania, 1997

[80] Russo I., Il Vallone Amara Nord a Monte Tauro Archeologia: cenni su contest, strutter, materiali. Natorziario storico di Augusta. 1998 21, 69–93

[81] Guzzardi L. Richerche archeologiche nel Siracusano. Kokalos. 1993–1994 39–40: 1299–1307.

[82] Bernabo Brea L., La cueva Corrugi en el territorio de Pachino, Ampurias, 1949 11, 1–23

[83] Brea Bernabo. l. Grotte di Pedagaggi (Carlentini). Archeologia nella Sicilia Sud Orientale. 1973 17–8

[84] Di Geronimo I, Di Mauro E, Di Stefano I, Mangano G. Riparo sotto roccia a Pedagaggi (Siracusa) con industria dell’Epigravettiano finale. Bullettino di Paleontologia Italiana; 1981–1992 83, 9–26

[85] Guzzardi L. Grotte sepolcrali dell’area iblea fra il Neolitico e l’età del bronzo. From Cave to Dolmen: Ritual and symbolic aspects in the prehistory between Sciacca, Sicily and the central Mediterranean. 2014 Dec 31:221.

[86] Galili E, Nir Y, Vachtman D, Mart Y. Physical characteristics of the continental shelves of the East Mediterranean basin. Submerged landscapes of the European continental shelf: quaternary paleoenvironments. 2017 Jul 5:377–403.

[87] Ashton N, Lewis SG, De Groote I, Duffy SM, Bates M, Bates R, et al. Hominin footprints from early Pleistocene deposits at Happisburgh, UK. PLoS One. 2014 Feb 7;9(2): e88329. doi: 10.1371/journal.pone.0088329 24516637 PMC3917592

[88] Bayón MC, Politis GG. The inter-tidal zone Site of La Olla: early–Middle Holocene human adaptation on the Pampean coast of Argentina. Prehistoric archaeology on the continental shelf: a global review. 2014:115–30.

[89] Ogloblin Ramirez I, Dunseth ZC, Shalem D, Shahack‐Gross R. Infrared spectra of mixtures of heated and unheated clay: Solving an interpretational conundrum. Geoarchaeology. 2023 Nov;38(6):822–9.

[90] Patania I, Goldberg P, Cohen DJ, Yuan J, Wu X, Bar‐Yosef O. Micromorphological and FTIR analysis of the upper Paleolithic early pottery site of Yuchanyan cave, Hunan, South China. Geoarchaeology. 2020 Mar;35(2):143–63.

[91] Cabanes D., Weiner S., & Shahack-Gross R., Stability of phytoliths in the archaeological record: a dissolution study of modern and fossil phytoliths. Journal of Archaeological Science, 2011, 38(9), 2480–2490.

[92] Tomáŝková S. What is a burin? Typology, technology, and interregional comparison. Journal of Archaeological Method and Theory. 2005 Jun;12:79–115.

[93] Cerasoni JN. Vectorial application for the illustration of archaeological lithic artefacts using the “Stone Tools Illustrations with Vector Art”(STIVA) Method. PloS one. 2021 May 11;16(5): e0251466. doi: 10.1371/journal.pone.0251466 33975331 PMC8112888

[94] Vigliardi A. Gli strati paleo-mesolitici della Grotta di Levanzo: con uno studio sulla fauna di P. Cassoli ed A. Tagliacozzo. Rivista di Scienze Preistoriche. 1982(37):79–134.

[95] Aranguren BM, Revedin A. Il giacimento mesolitico di Perriere Sottano (Ramacca, CT). Bullettino di Paletnologia Italiana. 1998;89:31–72.

[96] Nicoletti Fabrizio. "L’industria del riparo di Cafici nella valle di Terrana, Caltagirone, Catania." L’industria del riparo di Cafici nella valle di Terrana, Caltagirone, Catania. 1999 105–125.

[97] Segre A., Vigliardi A. L’Epigravettiaen evolue et inal en Sicile, RSP, 1983, XXX–VIII, 12, 351–369

[98] Heffter E.M. Absence of Evidence or Evidence of Absence? Assessing the Intensity of Early Upper Paleolithic Occupations in Serbia and the Central Balkans. University of Arizona, 2021.

[99] Riel-Salvatore J, Barton CM. Late Pleistocene technology, economic behavior, and land-use dynamics in southern Italy. American antiquity. 2004 Apr;69(2):257–74.

