# Supplementary material for: Between land and sea: A multidisciplinary approach to understand the Early Occupation of Sicily (EOS)
Source: PLoS One. 2024 Oct 9;19(10):e0299118. doi: 10.1371/journal.pone.0299118 (PMC11463786; doi:10.1371/journal.pone.0299118)
Supplement: S1 Table — (DOCX) [file pone.0299118.s001.docx]

**Between land and sea: A multidisciplinary approach to understand the Early Occupation of Sicily (EOS).**

**Supporting Information**

**S1 Table: List of land sites identified during survey.**

| **EOS field code** | **Names found in the literature** | **Notes** | **Latitude** | **Longitude** |
| --- | --- | --- | --- | --- |
| Identified from bibliographic research | | | | |
| Acquasanta | Monte Amara Sud (Russo, 2002), Monte Amara (Mentesana, 1967) | Cave | 37.27275 | 15.22044444 |
| Campolato Sud B II | Vallone Amara Nord grotta 3 (Russo 1998), Vallone Amara grotta 2 (Russo 2002), Capo Campolato BII (Guzzardi 1993-1994) | Cave | 37.27802778 | 15.21555556 |
| Campolato Sud B I | Vallone Amara Nord (Lanteri 1996), Vallone Amara nord 1-2 (Russo 1998), Vallone Amara nord 1-3 (Russo 2002), Capo Campolato BI (Guzzardi, Isacco not published) | Cave | 37.27813889 | 15.21597222 |
| VAN cave 4 | Vallone Amara Nord grotta 4 | Cave | 37.27852778 | 15.21583333 |
| VAN cave 5 | Vallone Amara Nord grotta 5 | Cave | 37.27863889 | 15.21580556 |
| Campolato Sud A | Vallone Amara Nord grotta 6 (Russo, 2002), Campolato Sud A (Guzzardi, 1993-1994) | Cave | 37.27872222 | 15.21611111 |
| Campolato cave 4 | Campolato grotte (Russo 2002), (drawn Russo/Giannino 1987), Campolato 37 (Lanteri 1997) | Cave | 37.28197222 | 15.21158333 |
| Campolato cave 1 | Campolato grotta 1(Russo 2002), (drawn Russo/Giannino 1987), Campolato 37 (Lanteri 1997) | Cave | 37.28163889 | 15.21180556 |
| Campolato RockShelter | Campolato rockshelter (riparo sottoroccia)  (Russo 2002), (Russo/Giannino 1987), (Lanteri 1997) | Rockshelter | 37.28136111 | 15.213 |
| Campolato cave 3 | Campolato grotta above the rockshelter (sovrastante riparo sottoroccia) (Russo/Giannino 1987), Campolato 37 (Lanteri 1997) | Cave | 37.28125 | 15.21358333 |
| Campolato 2 | Campolato 2 (Russo 2002), Campolato 36 (Lanteri 1997) |  | 37.285605 | 15.209553 |
| Cozzo Telegrafo | Russo (2002) | Cave system with WWII bunker | 37.28988889 | 15.14127778 |
| Vallone Maccaudo | Russo (2002) |  | 37.28461111 | 15.14180556 |
| Corruggi | Corruggi (Bernabo Brea 1949) |  | 36.7124341 | 15.1206107 |
| New Finds |  |  |  |  |
| A | ID by boat | Cave on land to be checked | 37.26741667 | 15.22863889 |
| B | ID by boat | Cave on land to be checked | 37.26430556 | 15.23344444 |
| C - Grotta del monaco | Local folklore | Cave with stalagmites outside WWII artillery posts | 37.23425 | 15.25311111 |
| Profile | ID during land survey | Geological profile cut by Gelso river, Pedagaggi | 37.19619444 | 14.96533333 |
| Cave 1 | ID during land survey | Large cave visible from the road, squared entrance, Pedagaggi | 37.19544444 | 14.96361111 |
| Rockshelter 1 | ID during land survey | Rockshelter in Pedagaggi | 37.19547222 | 14.96361111 |
| Twin Caves | ID during land survey | Two caves adjacent to each other, Padagaggi | 37.19541667 | 14.96311111 |
| Cave 2 | ID during land survey | Large Cave visible from the road, squared entrance, Pedagaggi | 37.19544444 | 14.96305556 |
| Cave 3 | ID during land survey | Cave with round external wall made of big stone, vestibule, rounded room and animal bones | 37.19547222 | 14.96283333 |
| Rockshelter 2 | ID during land survey | Rockshelter Pedagaggi | 37.19541667 | 14.96277778 |
| VAN RS1 | ID during land survey | Rockshelter | 37.27780556 | 15.21533333 |
| VAN RS2 | ID during land survey | Rockshelter | 37.27772222 | 15.21522222 |
| VAN RS3 | ID during land survey | Rockshelter | 37.27758333 |  |
