# Supplementary material for: Between land and sea: A multidisciplinary approach to understand the Early Occupation of Sicily (EOS)
Source: PLoS One. 2024 Oct 9;19(10):e0299118. doi: 10.1371/journal.pone.0299118 (PMC11463786; doi:10.1371/journal.pone.0299118)
Supplement: S2 Table — Submerged caves and paleosols were found during our boat and underwater surveys. (DOCX) [file pone.0299118.s002.docx]

**Between land and sea: A multidisciplinary approach to understand the Early Occupation of Sicily (EOS).**

**Supporting Information**

**S2 Table: List of underwater caves and clay deposits.** Submerged caves and paleosols were found during our boat and underwater surveys.

| **EOS field code** | **Description** | **Coordinates** |
| --- | --- | --- |
| Cave 1 | Cave in the cliff fill with stones | 37º14'02.0**"**N 15º14'59.2**"**E |
| Cave 2 | Cave in the cliff fill with stones | 37º14'02.5**"**N 15º15'01.2**"**E |
| Cave 3 | Cave in the cliff fill with stone and pottery | 37°14'01.0"N 15°15'01.0"E |
| Cave 4 | Cave in the cliff fill with stone and pottery | 37°14'01.0"N 15°15'01.0"E |
| Cave 5 | Cave in the cliff fill with stone, fine sand and pottery (Neolithic) | 37°14'01.0"N 15°15'01.0"E |
| Cave 6 | Underwater cave fill with stones | 37°14'54.2"N 15°14'41.2"E |
| Cave 7 | Underwater cave fill with stones | 37°14'54.2"N 15°14'41.2"E |
| Cave 8 | Cave in the cliff fill with stone | 37°14'54.2"N 15°14'41.2"E |
| Cave 9 + clay | Cave in the cliff fill with stone and pottery. Clay is reported in this location | 37°14'54.2"N 15°14'41.2"E |
| Cave 10 | Cave in the cliff fill with stones | 37°15'03.0"N 15°14'42.6"E |
| Cave 11 | Cave in the cliff fill with stones | 37°15'03.0"N 15°14'42.6"E |
| Grotta della Seggia | (Von Adrian 1978)  Cave in cliff with dry chambers preserving Middle Pleistocene fauna and Neolithic pottery | 37°05'49.1"N 15°18'03.4"E |
| Clay 1 | Underwater grey clay deposits sampled | 37°15'11.2"N 15°14'40.0"E |
