# Supplementary material for: Between land and sea: A multidisciplinary approach to understand the Early Occupation of Sicily (EOS)
Source: PLoS One. 2024 Oct 9;19(10):e0299118. doi: 10.1371/journal.pone.0299118 (PMC11463786; doi:10.1371/journal.pone.0299118)
Supplement: S3 Table — Mineralogical composition was determined by FTIR spectroscopy: Cl = Clay, (?) = Possible altered clay, (u/a) = unaltered by heat, Qz = quartz, Ca = Calcite, CHAP = carbonate hydroxylapatite. Phytoliths quantification are expressed in million per gram of sediment. (DOCX) [file pone.0299118.s003.docx]

**Between land and sea: A multidisciplinary approach to understand the Early Occupation of Sicily (EOS).**

**Supporting Information**

**S3 Table: List of sediment samples from Grotta della Seggia.** Table shows the results of bulk analyses from the first chamber of that cave. Mineralogical composition was determined by FTIR spectroscopy: Cl = Clay, (?) = Possible altered clay, (u/a) = unaltered by heat, Qz = quartz, Ca = Calcite, CHAP = carbonate hydroxylapatite. Phytoliths quantification are expressed in million per gram of sediment.*.*

| **Sample number** | **Stratigraphic layer** | **Description** | **Mineralogy** | **Phytoliths** |
| --- | --- | --- | --- | --- |
| GS-1 | 4 | Dark Brown silty sand sediment | Cl(a/u), Qz (-) | 0.30 millions per gram |
| GS-2 | 3 | Red sandy clay sediment | Cl(a/u), Qz (-) | 1.00 millions per gram |
| GS-3a | 2 | Black compacted sandy sediment rich in organic matter | Cl(?), CHAP, Qz (-) | 0.60 millions per gram |
| GS-3b | 1 | Red sandy clay sediment | Cl(?), CHAP, Qz (-) | 0.40 millions per gram |
| GS-3c | 1 | Red sandy clay sediment rich in bone | Cl(a/u), Ca, Qz (-) | 0.20 millions per gram |
