# Supplementary material for: Between land and sea: A multidisciplinary approach to understand the Early Occupation of Sicily (EOS)
Source: PLoS One. 2024 Oct 9;19(10):e0299118. doi: 10.1371/journal.pone.0299118 (PMC11463786; doi:10.1371/journal.pone.0299118)
Supplement: S4 Table — (DOCX) [file pone.0299118.s004.docx]

**Between land and sea: A multidisciplinary approach to understand the Early Occupation of Sicily (EOS).**

**Supporting Information**

**S4 Table: List of beach depositis used to quantify uplift since MIS5e**

| **EOS field code** | **Description** | **Latitude** | **Longitude** |
| --- | --- | --- | --- |
| Beach 1 | Beach deposits with e. Mnadriensis fossils | 37.281393, | 15.213715 |
| Beach 2 | Cemented micro-fossils | 37.268327, | 15.227948 |
| Beach 3 | Beach with Strombus bubonius | 37.267997, | 15.228634 |
| Beach 4 | Beach with Strombus bubonius | 37.268390, | 15.227282 |
| Beach 5 | Beach with Strombus bubonius | 37.272291, | 15.222263 |
| Other deposits | | | |
| Acquasanta 6 | Cementation of pebbles | 37.268327, | 15.227567 |
